# The Loss of Expression of a Single Type 3 Effector (CT622) Strongly Reduces *Chlamydia trachomatis* Infectivity and Growth

**DOI:** 10.3389/fcimb.2018.00145

**Published:** 2018-05-15

**Authors:** Mathilde M. Cossé, Michael L. Barta, Derek J. Fisher, Lena K. Oesterlin, Béatrice Niragire, Stéphanie Perrinet, Gaël A. Millot, P. Scott Hefty, Agathe Subtil

**Affiliations:** ^1^Unité de Biologie Cellulaire de l'Infection Microbienne, Institut Pasteur, Paris, France; ^2^Centre National de la Recherche Scientifique UMR3691, Paris, France; ^3^Collège Doctoral, Sorbonne Université, Paris, France; ^4^Department of Molecular Biosciences, University of Kansas, Lawrence, KS, United States; ^5^Department of Microbiology, Southern Illinois University, Carbondale, IL, United States; ^6^Institut Curie, PSL Research University, Centre National de la Recherche Scientifique UMR 144, Molecular Mechanisms of Intracellular Transport, Paris, France; ^7^Institut Pasteur—Bioinformatics and Biostatistics Hub—C3BI, USR3756 IP Centre National de la Recherche Scientifique, Paris, France

**Keywords:** host-pathogen interactions, effector proteins, genetic manipulation, *Chlamydia trachomatis*, chaperone proteins, structural biology, prenylation

## Abstract

Invasion of epithelial cells by the obligate intracellular bacterium *Chlamydia trachomatis* results in its enclosure inside a membrane-bound compartment termed an inclusion. The bacterium quickly begins manipulating interactions between host intracellular trafficking and the inclusion interface, diverging from the endocytic pathway and escaping lysosomal fusion. We have identified a previously uncharacterized protein, CT622, unique to the *Chlamydiaceae*, in the absence of which most bacteria failed to establish a successful infection. CT622 is abundant in the infectious form of the bacteria, in which it associates with CT635, a putative novel chaperone protein. We show that CT622 is translocated into the host cytoplasm via type three secretion throughout the developmental cycle of the bacteria. Two separate domains of roughly equal size have been identified within CT622 and a 1.9 Å crystal structure of the C-terminal domain has been determined. Genetic disruption of *ct622* expression resulted in a strong bacterial growth defect, which was due to deficiencies in proliferation and in the generation of infectious bacteria. Our results converge to identify CT622 as a secreted protein that plays multiple and crucial roles in the initiation and support of the *C. trachomatis* growth cycle. They reveal that genetic disruption of a single effector can deeply affect bacterial fitness.

## Introduction

*Chlamydia trachomatis* is the most common sexually transmitted bacterial pathogen. Infections of the urogenital mucosae are often asymptomatic but can lead to severe pathologies including pelvic inflammatory disease, ectopic pregnancy, and infertility (Brunham and Rey-Ladino, 2005). Other serovars are able to colonize the eye conjunctiva: repeated infections by *C. trachomatis* and subsequent corneal scarring is the leading cause of blindness by an infectious agent (Taylor et al., 2014). Overall, *C. trachomatis* infections have a profound impact on public health.

*C. trachomatis* are obligate intracellular bacteria. They undergo a characteristic bi-phasic developmental cycle, which typically completes within ~48 h (reviewed in AbdelRahman and Belland, 2005). The infectious form, called the elementary body (EB), predominantly infects epithelial cells of the genital tract or conjunctiva. Once internalized, the bacteria reside in a parasitophorous vacuole referred to as an inclusion. Within this enclosed space, EBs convert to larger, replicative reticulate bodies (RBs). After several rounds of division, RBs asynchronously convert back to the infectious form, before ultimately exiting the host cell.

Throughout this cycle, the organism manipulates the host in order to penetrate into the cell, escape innate defense mechanisms, acquire nutrients, and multiply steadily. Many of the underlying molecular mechanisms responsible for host manipulation are still poorly understood. *C. trachomatis* devotes a large proportion of its small genome (encoding only about 900 proteins) to the synthesis of proteins translocated into the host cytoplasm, where they hijack key cellular pathways and host resources. Protein translocation is mainly achieved by type 3 secretion (T3S). This secretion system is shared by many Gram-negative bacteria that interact with eukaryotic cells and directs the translocation of so-called bacterial effectors across both bacterial membranes, and a third eukaryotic membrane (Galán et al., 2014). T3S machineries have been extensively studied in genetically tractable bacteria, and most of their characteristics are conserved in chlamydiae (Ferrell and Fields, 2016). In particular, many effectors require ancillary chaperone proteins for efficient translocation. These chaperones share common features such as a small size, an acidic isoelectric point, and a stable homodimer conformation (Thomas et al., 2012). In contrast, the effector proteins are often pathogen-specific with a limited degree of conservation at the amino acid level, often making their identification difficult.

EBs are primed with a battery of T3S complexes and with stored effectors that have been synthesized at the end of the previous developmental cycle (Cossé et al., 2016). So far only four effectors originating within the *C. trachomatis* EB have been characterized. The Translocated actin-recruiting phosphoprotein (TarP) is a multi-domain scaffold protein that interacts with the actin cytoskeleton at several levels, either directly via its intrinsic actin nucleation activity or indirectly by engaging actin or actin binding proteins (reviewed in Cossé et al., 2016). Another effector involved in invasion is CT694/TmeA (McKuen et al., 2017). Like TarP, the Translocated early phosphoprotein TepP is an immediate effector phosphorylated on tyrosine upon translocation into the host cytoplasm, allowing its interaction with the scaffolding protein CrkI/II. Cells infected with a *tepP* mutant showed altered expression of a subset of genes associated with the innate immune response, suggesting that this effector may serve to interfere with signaling cascades important for the regulation of the innate immune response to *Chlamydia* (Chen et al., 2014; Carpenter et al., 2017). Finally, CT695/TmeB is another immediate effector of unknown function (Mueller and Fields, 2015). Intriguingly, TarP, TmeA, TmeB, and TepP share the same T3S chaperone, Slc1 (Brinkworth et al., 2011; Pais et al., 2013; Chen et al., 2014).

It is likely that EBs are packed with several other “immediate” effectors to support the invasive process as well as the protection of the nascent inclusion before translocation of a second wave of newly-synthesized effectors upon EB to RB conversion (Cossé et al., 2016). To identify those immediate effectors, we searched the literature for hypothetical proteins highly enriched in EBs, and which either possessed a functional T3S signal (Subtil et al., 2005; da Cunha et al., 2014), or had been detected in the host cytosol. In this report, we show the hypothetical protein CT622, which had previously been detected in the host cell cytosol (Gong et al., 2011) is a T3S effector and we identify a novel putative chaperone protein for this effector. Furthermore, we show that genetic disruption of *ct622* deeply affects bacterial infectivity and growth *in vitro*.

## Results

### CT622 is a T3S effector and binds to CT635 through its N-terminus

**Figure 1:**
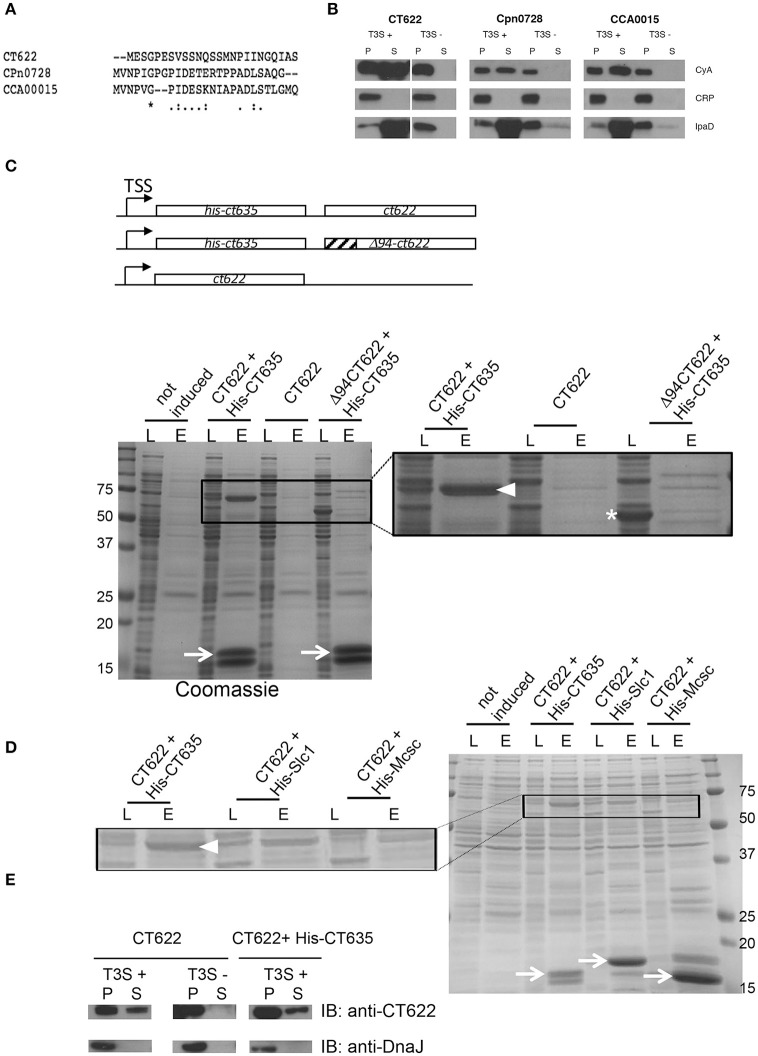
CT622 and its homologs are T3S substrates. **(A)** The first 25 amino acids of CT622 and its homologs in *C. pneumoniae* (CPn0728) and *C. caviae* (CCA00015) are shown. **(B)** The amino-terminal segments of the indicated proteins were fused to the Cya reporter protein and expressed in a *S. flexneri ipaB* (constitutive T3S) or *mxiD* (defective T3S) strain. Exponential phase cultures expressing the reporter fusion protein were fractionated into supernatants (S) and pellets (P). Samples were resolved by SDS-PAGE, transferred to a PVDF membrane, and probed with anti-Cya (to detect chlamydial fusion proteins), anti-IpaD (*Shigella* secreted protein), or anti-CRP (*Shigella* non-secreted protein) antibodies. **(C)** Top: schematic view of the plasmids used. Bottom: CT622 co-purify with CT635. *E. coli* were transformed with a plasmid expressing CT622 and HIS-CT635 (lanes 1–4), CT622 alone (lanes 5, 6), or CT622 truncated of 94 amino acids and HIS-CT635 (lanes 7,8). HIS-tagged proteins were pulled-down from bacterial lysates using nickel-coupled beads. Whole bacterial lysates (L) and eluted fractions (E) are shown. Samples were resolved by SDS-PAGE and strained with Coomassie blue, arrows point to the HIS-tagged chaperone (expected mw = 18 kDa). The inset shows a two-fold enlargement, with arrowhead pointing to CT622 in the elution fraction when co-expressed with HIS-CT635, and the asterisk to Δ94CT622. **(D)** Same as in C, with *E. coli* transformed with a plasmid expressing CT622 and His-CT635 (lanes 1–4), CT622 and His-Slc1 (lanes 5, 6), or CT622 and His-Mcsc (lanes 7,8). Arrows point to the His-tagged proteins, arrowhead to CT622. The inset shows a two-fold enlargement. **(E)** The indicated *S. flexneri* strains were transformed with a plasmid expressing CT622 alone or together with HIS-CT635. Translocation of CT622 in the culture supernatant (S) was analyzed as described in **(B)**, with the exception that antibodies against DnaJ were used to control for bacterial lysis.

The majority of secreted chlamydial proteins are translocated via the T3S machinery. To explore if this was also true for CT622, we first tested for the presence of a functional T3S signal within the N-terminus using a heterologous secretion assay in *Shigella flexneri* as described previously (Subtil et al., 2001). We also tested orthologs of CT622 in *C. pneumoniae* (CPn0728) and *C. caviae* (CCA00015) because the 25 first amino acids of the three proteins largely differ (Figure 1A). Briefly, we fused these N-terminal sequences to the reporter protein calmodulin-dependent adenylate cyclase (Cya). The chimeric proteins were then expressed in *S. flexneri ipaB* (constitutive T3S) or *mxiD* (deficient in T3S) null strains (Allaoui et al., 1993; Ménard et al., 1993). When we analyzed the culture supernatant versus the bacterial pellet, we found the Cya reporter in the supernatant of *ipaB* cultures (Figure 1B). The same expression pattern was observed for an endogenous *Shigella* T3S substrate, IpaD (Ménard et al., 1993). Conversely, we found the cAMP receptor protein (CRP), a non-secreted protein, exclusively in the bacterial pellet. This control shows that Cya detection in the supernatant was not due to bacterial lysis. Finally, the Cya reporter was not recovered in the supernatants of the *mxiD* strain, demonstrating that its secretion was dependent on T3S. Altogether, these results demonstrate that the chimeric proteins were translocated via a T3S-dependent mechanism. As there is little conservation in the N-terminal extremity of the three orthologs, the finding that all three sequences function as T3S signals strongly support the hypothesis that the corresponding proteins are T3S effectors.

Many T3S effectors are bound to chaperone proteins prior to secretion. To test if CT622 could interact with a bacterial protein we performed a pull-down on chlamydial EB lysates using GST-tagged proteins as bait. Proteins retained on the glutathione columns were identified by mass spectrometry and were considered as potential hits if pulled-down with GST-CT622, and not with GST alone. The experiment was performed twice, and two proteins were substantially represented in both lists, MOMP and CT635 (Table S1). MOMP is the major outer membrane protein of *Chlamydia* and is a usual suspect for contamination as it is the most abundant protein in EBs. CT635 is a hypothetical protein of 17 kDa with a slightly acidic pI (6.5). To confirm its ability to bind CT622, we first obtained and purified a rabbit polyclonal antibody against CT622. This antibody recognized a protein of expected molecular weight (68 kDa) in lysates of *C. trachomatis* L2 infected cells that accumulated at late times of infection, consistent with published data (Gong et al., 2011; Figure S1A). Despite a stringent purification procedure, an additional minor product around 78 kDa was consistently observed by western blot. Host cells infected for 24 h were stained with anti-CT622 antibodies, which predominantly recognized organisms within the inclusion (Figure S1B). Pre-incubation of the antibody with GST-CT622 displaced most of the signal, while pre-incubation with an unrelated GST-tagged protein did not. In the presence of an excess of GST-CT622 a weak signal was however still visible in the inclusion that likely corresponds to the bacterial cross-reaction product of 78 kDa detected by western blot.

In order to provide orthogonal support for the putative CT622-CT635 interaction, we co-expressed CT635 with a N-terminal histidine tag together with CT622 in *Escherichia coli*. CT622 co-purified on a nickel column only in the presence of HIS-CT635 (Figure 1C). T3S chaperones commonly bind the N-terminal region of their cognate effector, usually within the first 100 amino acids (Parsot et al., 2003). Analysis of the CT622 N-terminus with PSI-PRED (Buchan et al., 2013) predicted a disordered domain within the first ~94 amino acids of CT622. When we expressed a deletion mutant of CT622 truncated of its first 94 amino acids, it did not co-purify with HIS-CT635 (Figure 1C). These observations support that the N-terminus of CT622 is crucial for the interaction with CT635. To know if other chlamydial chaperones were also able to interact with CT622 we co-expressed CT622 with His-tagged Slc1 or Mcsc, two other chaperones for the export of T3S substrates in *C. trachomatis* (Spaeth et al., 2009; Brinkworth et al., 2011; Saka et al., 2011). CT622 did not co-purify with His-Mcsc, and co-purified with His-Slc1 to a lesser extent than with His-CT635 (Figure 1D).

Finally, we tested if CT622 as a full-length protein could be secreted by *S. flexneri*. We first transformed the *S. flexneri* T3S competent and defective strains (*ipaB* and *mxiD*, respectively) with a plasmid expressing CT622 alone (Figure 1E, left). A fraction of CT622 was detected in the culture supernatant of the T3S competent strain and not of the T3S defective one, indicating that the T3S machinery of *S. flexneri* can export CT622, although not very efficiently. Co-expression of HIS-CT635 in the T3S competent strain did not significantly increase the proportion of protein that was recovered in the culture supernatant (Figure 1E, right). Thus, although CT635 shows several properties of T3S chaperone proteins, it remains to be determined if it enhances CT622 secretion in *C. trachomatis*, as it does not appear to do so in the surrogate host *S. flexneri*.

### The C-terminal domain of CT622 displays structural similarities with geranylgeranyl transferases and synthases

In order to gain more insights into the molecular mechanism of CT622 function during *Chlamydia* infection, we undertook a structural analysis of CTL0886, the CT622 ortholog in the strain *C. trachomatis* L2 434/Bu. The two proteins show a higher degree of variation than expected (89% sequence identity, Figure S2) as there is >99% conservation amongst each genome at the nucleotide level (Thomson et al., 2008). Intriguingly, this divergence is clustered within their N-terminal regions (82% identity, residues 1–352) as the C-terminal regions share >98% identity. Nonetheless, to avoid confusion, the serovar L2 434/Bu nomenclature (i.e., CTL0886) will be used for all the structural data and amino acid positions specific to this sequence are reported.

A construct lacking the disordered N-terminus (residues 94–651, CTL0886^FL^) was recombinantly expressed and purified (Figure S3). During purification and subsequent storage at 4°C, CTL0886^FL^ appeared to degrade into a ~25 kDa fragment, however, closer inspection indicated that a doublet was present within the SDS-PAGE gel (two arrows in Figure S3). Indeed, LC-MS/MS analysis (data not shown) of the excised bands suggested that two unique CTL0886^FL^ domains were present spanning residues 94–334 (CTL0886^N^) and residues 353–651 (CTL0886^C^). Each of these regions were recombinantly expressed (CTL0886^N^ was truncated to residues 94–322 according to secondary structure predictions), purified to homogeneity and screened for crystallization. Single bipyramidal crystals of CTL0886^N^ were obtained, but diffraction of synchrotron X-rays was limited to ~6 Å resolution (data not shown). Despite extensive optimization, crystals that diffracted to higher resolution were not obtained.

**Figure 2:**
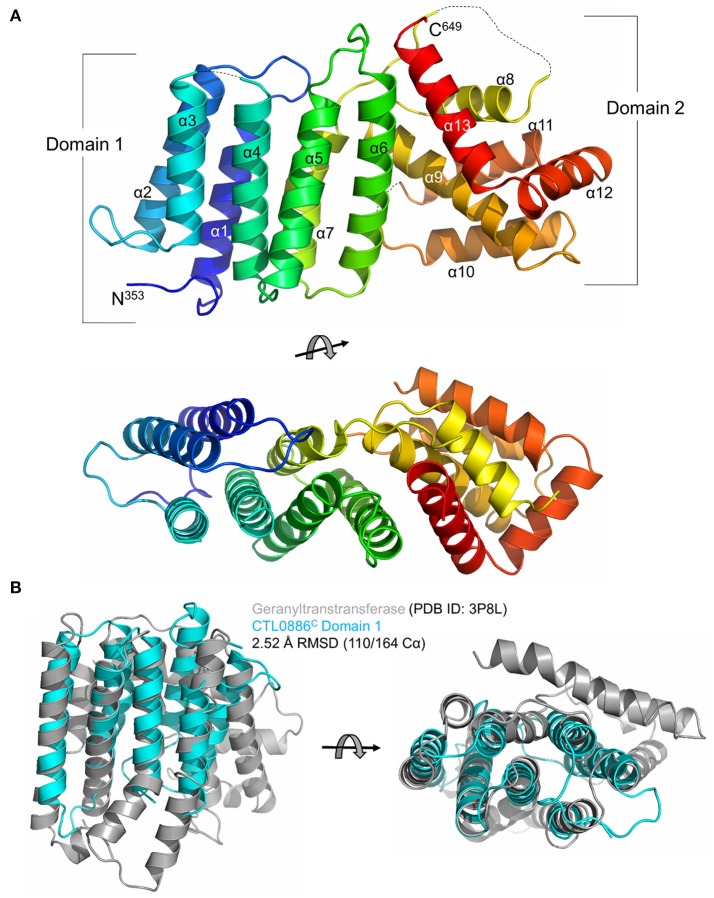
Structure of the C-terminal domain of CTL0886. **(A)** Crystal structure of CTL0886^C^ is shown in cartoon ribbon format using common rainbow colors (slowly changing from blue N-terminus to red C-terminus). Secondary structure lacking interpretable electron density is shown as dashed line*. Two* apparent domains are labeled, with domain 1 comprising α1-7 and domain 2 comprising α8-13. Structure is rotated 90° about the horizontal axis below. **(B)** Cartoon ribbon diagram of a structural alignment of domain 1 from CTL0886^C^ (residues 353-517, cyan) and GGTase from *E. faecalis* (PDB ID: 3P8L, gray) with an RMSD of 2.52 Å over 110/164 Cα atoms within 5.0 Å, rotated 90° about horizontal axis.

While equally poor diffraction was initially obtained with CTL0886^C^ crystals, reductive methylation of surface exposed lysines (Walter et al., 2006) enabled the growth of cubic crystals that diffracted synchrotron X-rays to 1.90 Å resolution (Table S2). The structure of CTL0886^C^ was determined using selenomethionine-labeled recombinant protein (also dimethylated) by Se-SAD and refined against the native high-resolution dataset. The final model is comprised of a single polypeptide in the asymmetric unit, 1 sulfate anion and 98 water molecules. The CTL0886^C^ structure is comprised of 13 α-helices spanning two separate domains (Figure 2A). Domain 1 consists of helices α1–7 arranged in a 7 helix bundle (residues 353–517). Helices α8–13 of domain 2 adopt an intertwined, globular fold with the later 3 helices (α11–13) wrapping around helices α8–10. Upon inspection of the 2*F*_*o*_-*F*_*c*_ electron density map, four lysine residues (446, 463, 490, and 579) were modeled as dimethyl-lysine (Rocchia et al., 2002; Sledz et al., 2010).

In order to identify functionally characterized proteins that share a structural fold with CTL0886^C^, the PDB was queried with the protein structure homology server DALI (Holm et al., 2006; Holm and Rosenstrom, 2010). Searches with the individual domains produced unique hits with higher Z-scores than intact CTL0886^C^ and are listed in Table S3. For domain 1, each of these structures (as well as the next 10 scoring hits) are all members of the polyprenyl transferase (PTS) family. Enzymes in this family typically catalyze condensation reactions involving isoprene units, providing substrates toward the biosynthesis of nearly all known isoprenoid metabolites (Kellogg and Poulter, 1997). Structural superposition of CTL0886^C^ domain 1 and geranylgeranyl transferase (GGTase) from *Enterococcus faecalis* (PDB ID: 3P8L, Wallrapp et al., 2013) indicates these proteins align with an RMSD of 2.52 Å across 110/164 Cα atoms (Figure 2B) despite sharing only 5% sequence identity. GGTases transfer geranylgeranyl (GG), a 20-carbon lipophilic chain, to C-terminal cysteine residues of specific proteins (Vandermoten et al., 2009). The most striking similarity between these two structures is the highly conserved spatial organization of each α-helix within the bundle. CTL0886^C^ domain 1 is, however, lacking several α-helices typically found within the PTS family, including those involved in dimerization and extended regions typically found within the active site of PTS family enzymes (e.g., Asp-rich motifs). Intriguingly, CT622 and its orthologs in *Chlamydiaceae* encode a region of Asp/Glu residues (red bar in Figure S2). Within the CTL0886^C^ structure this motif is found within a region of random coil prior to α1. It is currently unclear, given the lack of structural information for the remainder of the protein, how this motif is structured in the full-length protein. To test if the structural similarity with GGTase reflected an ability for CT622 to catalyze, or to inhibit, geranylgeranylation, we performed GGTase reactions *in vitro* using the small GTPase, Rab6, as a substrate. Purified GST-CT622 did not exhibit GGTase activity and did not interfere with the ability of purified human GGTase to prenylate Rab6 (Figure S4A). Furthermore, we did not detect an interaction between CT622 and NDB-farnesyl, a surrogate substrate for GGTases (Figures S4B,C). Thus, while the structural similarity between CT622 and GG transferases/synthases remains intriguing, further investigations will be needed to determine if it reflects a functional parenthood with this family of enzymes.

### Insertional disruption of *ct622* expression and strain complementation

**Figure 3:**
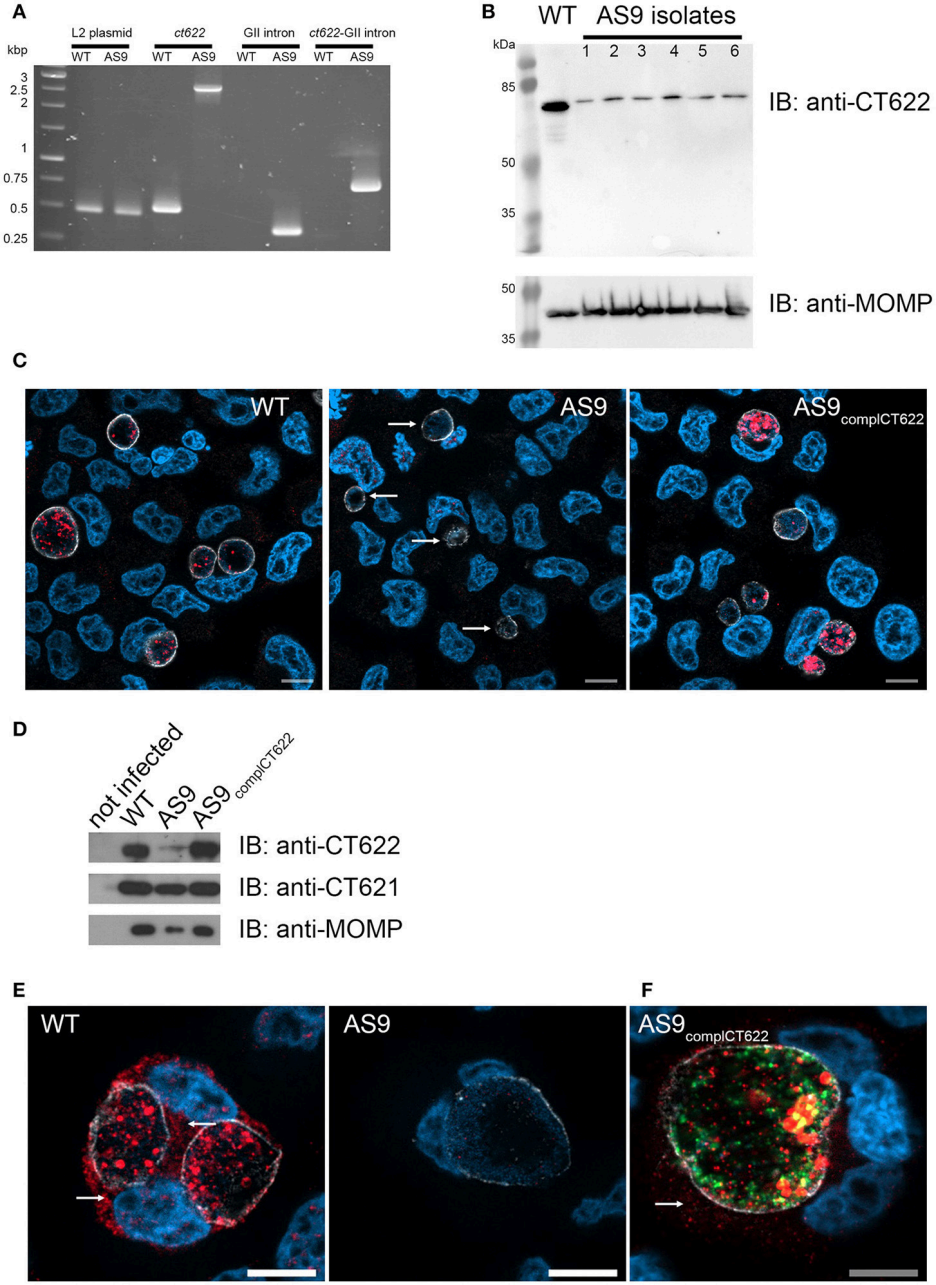
Loss of *ct622* expression due to intron insertion and complementation. **(A)** PCR verification of plaque purified *C. trachomatis* L2 *ctl0886*::GII(*aadA*) clones. PCR was performed using genomic DNA from wild type L2 (ACE051 strain) and the plaque purified AS9 clones to assess intron insertion, loss of the intron donor plasmid, and maintenance of the chlamydial cryptic plasmid. Primers were designed to amplify regions of the cryptic plasmid, *ctl0886* locus, intron, and to verify the orientation of the intron. Products were separated on 1% agarose gels and stained with ethidium bromide. The template used in the PCR reaction is listed at the top of each lane. The specificity of the primers used in each reaction is indicated above. Identical results were obtained for the 6 isolates, PCR products for one isolate are shown. **(B)** Crude EB preparations of each isolate and of the parental strain were analyzed by western blot using anti-CT622 (top), and anti-MOMP (bottom) antibodies. Note that only the contaminant 78 kDa band is still visible in the AS9 isolates. **(C)** Cells were infected for 30 h with wild type, AS9 or AS9_complCT622_ bacteria before fixation and permeabilization. Cells were stained with mouse antibodies against the inclusion protein CT813 and rabbit anti-CT622 antibodies followed with Cy3-conjugated anti-mouse (shown in white) and Alexa647-conjugated anti-rabbit (shown in red) antibodies. DNA was labeled with Hoechst 33342 (blue). Arrows point to inclusions in the AS9 infected cells. **(D)** Crude lysates of HeLa cells infected or not for 48 h with the indicated strains were analyzed by western blot using anti-CT622 (top), anti-CT621 (middle), and anti-MOMP (bottom) antibodies. **(E)** Cells infected for 48 h with wild-type (left) or AS9 (right) bacteria were stained with anti-CT813 and anti-CT622 antibodies followed with Cy3-conjugated anti-mouse (shown in white) and Alexa647-conjugated anti-rabbit (red) antibodies. DNA was labeled with Hoechst 33342. Arrows point to CT622 detected in the cytosol. **(F)** Cells infected for 48 h with AS9_complCT622_ bacteria were stained with mouse anti-flag antibodies and rabbit antibodies again the inclusion protein Cap1 followed with Alexa647-conjugated anti-mouse antibodies (red) and Cy3-conjugated anti-rabbit antibodies (white). Bacteria express GFP (green), DNA was labeled with Hoechst 33342 (blue). In panels **(E,F)**, arrows point to CT622 detected in the cytosol. Scale bar = 10 μm.

To study CT622 function during infection we attempted to generate a CT622 insertion mutant, a strategy that will only work if the gene is unessential for bacterial development in tissue culture. Global mapping of the transcription start sites in the *C. trachomatis* genome indicated that *ct622* has its own transcription starting site (Albrecht et al., 2010), making it a good candidate for single gene disruption. We applied the TargeTron^TM^ based strategy to disrupt *ctl0886* in *C. trachomatis* L2. We retargeted the intron in the plasmid pDFTT3 for *ctl0886* disruption, using *aadA* (encoding spectinomycin resistance) as a selection marker (further detailed in the Materials and Methods section). The resulting plasmid pTTmut9*aadA* was used to transform *C. trachomatis* L2. After 4 passages under spectinomycin selection, EBs were harvested and six clonal isolates were obtained. Six plaques were expanded and the isolates analyzed further. Primers flanking the intron insertion sites, as well as primers for intron amplification, were used to verify that the insertion had occurred at the targeted site, with the expected orientation. Maintenance of the cryptic *C. trachomatis* plasmid in the isolates was also verified (Figure 3A). Finally, bacteria were harvested at 48 h post infection (hpi), lysed and samples were run on SDS-PAGE to verify the absence of production of CT622 in each of the isolates using western blot (Figure 3B). Only the 78 kDa cross-reaction product was still visible in each of the isolates. The deletion mutant strain, designated as AS9, generated inclusions which were only weakly recognized by antibodies against CT622 (Figure 3C). The intensity of the signal was comparable to that measured in the parental strain in the presence of an excess of GST-CT622 (Figure S1) and is likely due to the cross-reaction signal detected by our antibody. Importantly, expression of *ct621*, which lies downstream of *ct622* (both genes are coded on the complementary strand), was normal in the AS9 strain (Figure 3D). The ability to obtain a deletion mutant of *ct622* shows that the gene is not absolutely required for *C. trachomatis* L2 development in cell culture.

Finally, we cloned *ctl0886* with its endogenous promoter, and a nucleotide sequence coding for a flag tag, immediately upstream of the stop codon into a plasmid also constitutively expressing GFP. The AS9 strain was stably transformed with this plasmid to generate a complemented strain designed as AS9_complCT622_. This strain expressed CT622 (Figure 3D), although the signal was not as homogenously distributed among bacteria as in the wild type strain (Figure 3C), likely reflecting heterogeneity in plasmid copy numbers among individual bacteria. At late times of infection (>48 hpi) our anti-CT622 polyclonal antibody detected a signal in the cytoplasm of cells infected with wild-type bacteria (Figure 3E). Specificity of the labeling was confirmed using anti-flag antibodies on cells infected with AS9_complCT622_ (Figure 3F).

### Loss of expression of *ct622* results in pleiotropic defects in *C. trachomatis* developmental cycle

**Figure 4:**
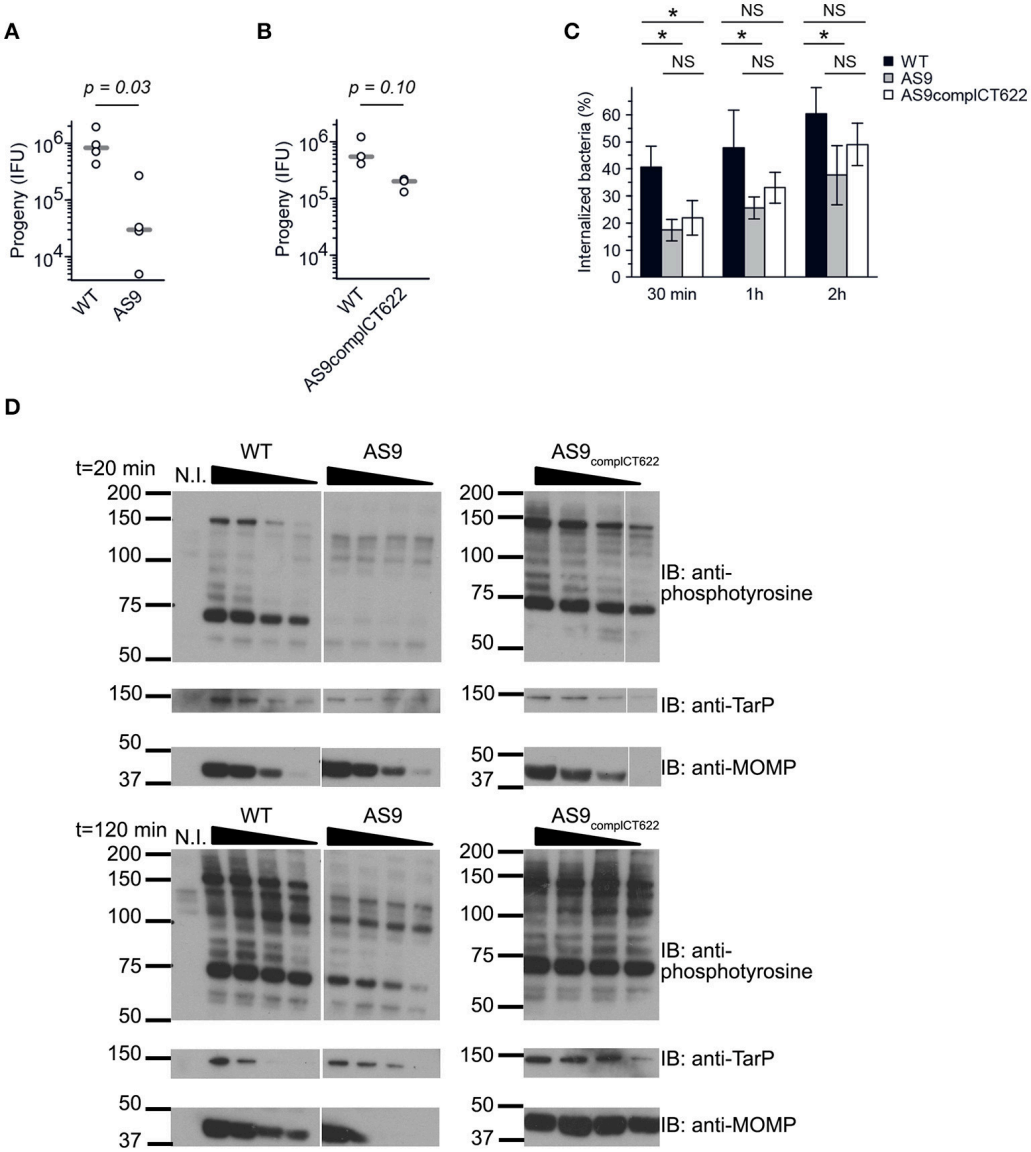
Knocking out CT622 expression impairs bacterial development. **(A,B)** Reinfectivity assays for the wild type L2 (ACE051 strain), AS9 and AS9_complCT622_ strains. Total progeny, normalized to an inoculum of 10^3^ IFU for each strain, was determined 48 hpi in a reinfection assay. Dot plot distribution of the progeny for 4 **(A)** and 3 **(B)** independent experiments is shown. Gray bar, median. The *p*-value comparing the two distributions is shown above (Mann–Whitney-Wilcoxon test). See Table S5 for all details on statistics. **(C)** Kinetics of entry. Cells were preincubated with bacteria (MOI = 10) for 30 min at 4°C before shifting the temperature to 37°C. At the indicated times, the samples were fixed and extracellular bacteria labeled with anti-LPS antibodies followed with Alexa647-coupled secondary antibodies (red). The samples were then permeabilized and intracellular bacteria were stained with anti-LPS antibodies followed with Alexa488-coupled secondary antibodies (green). Means ± standard deviation of 3 or 4 independent experiments are shown. The star indicates a significant difference of effect (contrast test) in the percentage of internalization; NS, no significant difference. **(D)** Tyrosine-phosphorylation pattern upon infection. Cells were incubated for 40 min at 4°C with increasing number of bacteria (two-fold increments) before shifting the plates to 37°C for 20 or 120 min. Crude lysates were analyzed by western blot with anti-phosphotyrosine antibody. Equivalence in terms of bacterial load for the three different strains used was verified with antibody against TarP and MOMP.

The AS9 strain was slow to expand, suggesting a potential growth defect. We tested this hypothesis by performing reinfection assays. Wild-type, AS9 and AS9_complCT622_ strains were titrated, and identical titers (inclusion forming units, IFUs) were used to infect fresh HeLa cell monolayers. The resulting progeny were collected 48 hpi and the number of IFUs collected was quantified by infecting new HeLa cells. We observed a 25-fold reduction in the progeny in the AS9 strain compared to the wild type strain (Figure 4A). Reintroduction of the disrupted gene on the plasmid partially restored the infectivity, as a 3.5-fold difference with the WT strain was observed (Figure 4B). Many of the AS9_complCT622_ bacteria did not express CT622 at a detectable level (Figures 3C,F), an observation that might explain the partial recovery in infectivity. Finally, transfecting cells with a plasmid expressing *ct622* prior to infection had no effect on AS9 infection (not shown), likely because ectopic expression did not reproduce CT622 spatio-temporal expression during infection.

Reduced levels of progeny formation by the AS9 strain could potentially reflect defects in a variety of different roles, including: early endocytic events, intracellular growth, and nutrient acquisition of RBs, the ability to differentiate into infectious EBs, and/or EB survival. We measured the ability of the bacteria to enter into cells by differential staining of intracellular versus extracellular bacteria. In the absence of CT622 bacterial entry was less efficient than in the parental strain, and after 2 h only about 35% of bacteria were internalized against 60% for the parental strain (Figure 4C). Entry efficiency was partially restored in the AS9 _complCT622_ strain.

We next looked at one of the earliest event that occurs upon infection, i.e. tyrosine phosphorylation of host proteins and of at least two effectors, TarP and TepP (Birkelund et al., 1994; Fawaz et al., 1997; Clifton et al., 2004; Chen et al., 2014). Infections were synchronized at 4°C with increasing numbers of WT, AS9, or AS9_complCT622_ bacteria, and shifted to 37°C for 20 or 120 min before cell lysis and analysis by western blot. Anti-MOMP and TarP antibodies were used to verify that bacterial load was comparable for the 3 different strains (Figure 4D). As expected, infection with wild-type bacteria resulted in tyrosine-phosphorylation of several proteins, including a prominent band around 75 kDa, and one migrating around 150 kDa, that corresponds to TarP (Clifton et al., 2004; Figure 4D). Infection-specific tyrosine phosphorylation was hardly detectable when cells were infected with AS9 bacteria and was recovered in the AS9_complCT622_ strain. TarP phosphorylation was still not detectable in cells infected with AS9 bacteria for 120 min, showing that absence of phosphorylation was not simply due to the delay in AS9 bacteria internalization.

### In the absence of *ct622* expression many bacteria fail to initiate a developmental cycle

**Figure 5:**
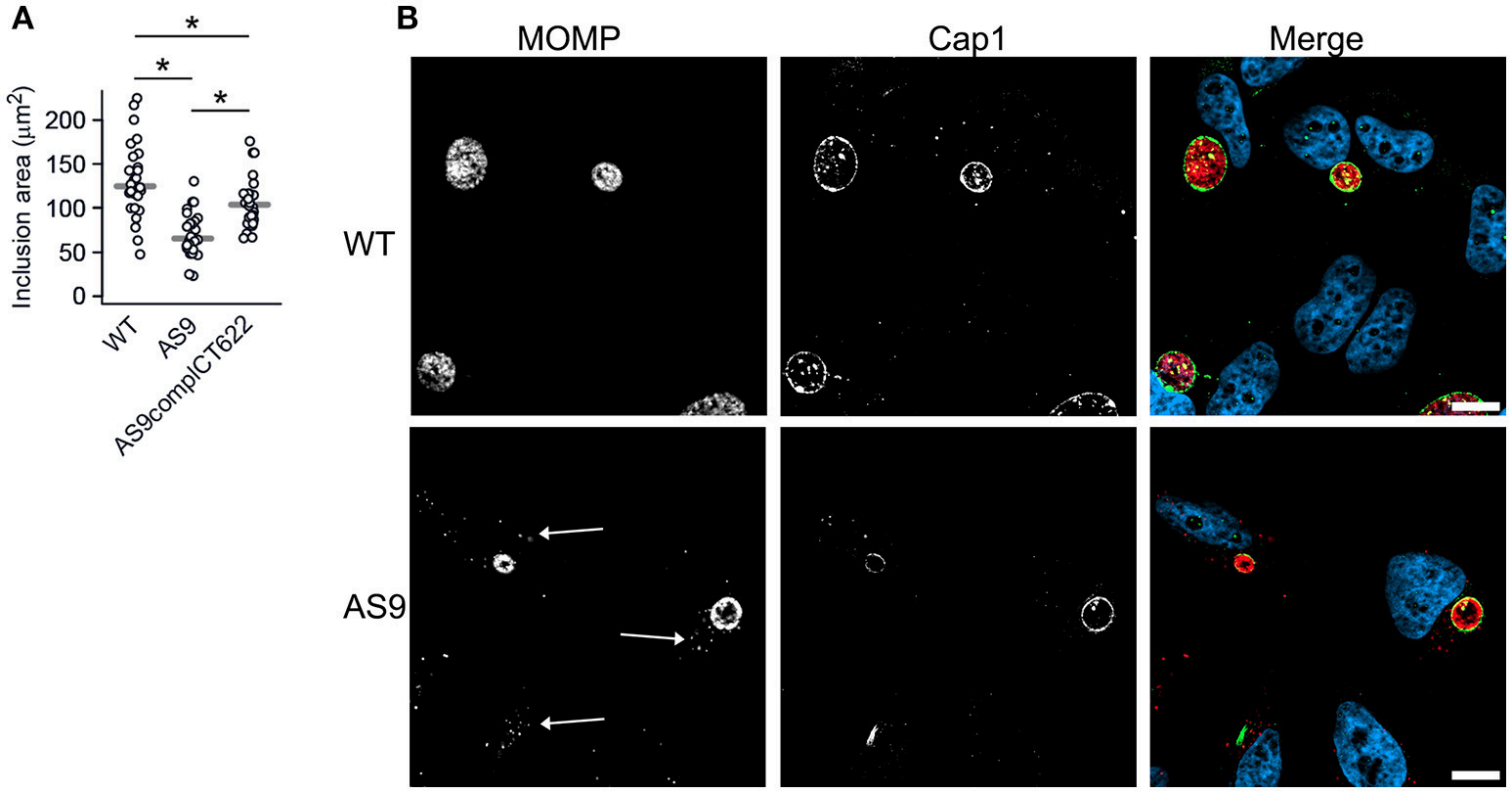
Knocking out CT622 expression impairs inclusion growth. **(A)** Dot plot distribution of the inclusion area in cells infected with WT, AS9, or AS9_complCT622_ bacteria for 24 h before fixation and DNA staining with Hoechst. Random fields were taken and the area of all inclusions in that field were measured using Zen software. Gray bar, median. The star indicates a significant difference (Mann-Whitney-Wilcoxon test). **(B)** Cells were incubated with wild type L2 (ACE051 strain) or AS9 for 24 h before fixation and permeabilization. Bacteria were stained with mouse anti-MOMP and rabbit anti-Cap1 antibodies followed with Cy5-conjugated anti-mouse and Alexa488-conjugated anti-rabbit antibodies. The merge images show nuclei in blue, the inclusion membrane (Cap1) in green and the bacteria (MOMP) in red. Scale bar = 10 μm.

The two-fold reduction in the entry efficiency does not account for the strong decrease in progeny measured 48 hpi, suggesting that the AS9 strain has other deficiencies. Cells infected for 24 h with the AS9 strains contained inclusions that had on average half the diameter of wild type inclusions, while the decrease was only of 17% in the complemented strain (Figure 5A, see also Figure 3C). Thus, the loss in progeny can also be partly explained by a slower growth rate. Except for their smaller size, AS9 inclusions had normal appearance and expressed the inclusion membrane marker Cap1 (Fling et al., 2001; Figure 5B). Most strikingly, MOMP staining revealed that cells infected with AS9 for 24 h also contained scattered bacteria. In contrast to the bacteria within inclusions, scattered bacteria were negative for Cap1 (Figure 5B).

**Figure 6:**
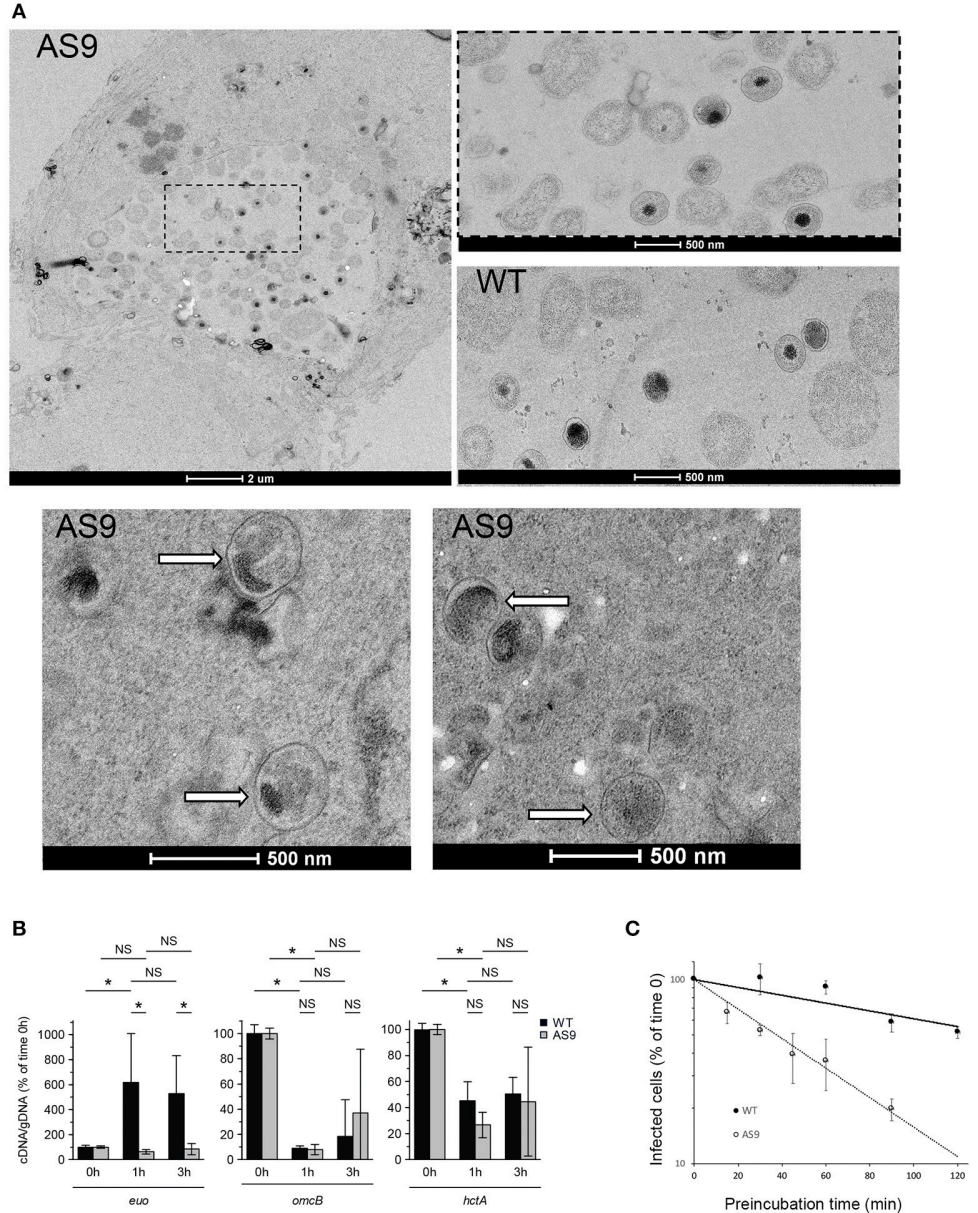
AS9 bacteria are impaired in their ability to initiate a developmental cycle. **(A)** HeLa cells were infected for 24 h, fixed and processed for transmission electron microscopy. AS9 infected cells displayed inclusions of normal appearance [top left panel. An enlargement of the boxed region is shown to the right, with below a view of an inclusion from cells infected with wild type (WT) bacteria for comparison]. In the two bottom panels arrows point to individual bacteria observed in the same preparation and which presented EB morphology. Such individual, EB-looking, bacteria were not observed in cells infected with the wild type strain. **(B)** Cells were infected for the indicated amount of time in duplicate wells for parallel RNA and DNA extractions. RNA was reverse transcribed and amounts of *euo, omcB*, and *hctA* cDNA and of genomic DNA (gDNA) were measured by qPCR for each time point. The bars show the means ± standard deviation of 3 independent experiments, each point measured in duplicates. The star indicates a significant difference of effect (contrast test) in relative mRNA levels; NS, no significant difference. **(C)** Bacteria were pre-incubated for the indicated time at 37°C in complete medium before being added to the cells. One day later cells were fixed and the percentage of infected cells was determined by flow cytometry. The percentage of infected cells for each pre-incubation time is plotted relative to the percentage of infection without pre-incubation. The mean of three independent experiments (±*SD*) is shown.

We examined these infected cells at the ultrastructural level. In some of the cells infected with the AS9 strain we observed regular inclusions, containing normal looking RBs, indistinguishable from wild type inclusions (Figure 6A). We also observed bacteria in individual vacuolar compartments that presented EB-like morphology, with a small size and condensed nucleoid. Such individual bacteria were never observed 24 hpi in cells infected with wild type bacteria. Thus, it appears that in the absence of CT622, while some bacteria manage to give rise to normal-looking, although smaller inclusions, a large proportion of the internalized bacteria fail to convert to the reticulate body form and to establish a niche suitable for intracellular growth. To test this hypothesis we looked at the level of transcription of *euo*, which is one of the first genes transcribed upon the initiation of the developmental cycle (Belland et al., 2003). As expected, we observed a sharp increase in the amount of *euo* transcripts in cells infected for 1 h with the wild type strain, while transcripts for the late genes *omcB* and *hctA* rapidly decreased. In contrast, *euo* transcription remained low in cells infected with AS9 bacteria. Late genes showed a similar profile as observed with the wild type strain (Figure 6B). The failure to initiate the EB to RB stage of the developmental cycle might result from the formation of defective EBs in the absence of CT622, and/or a rapid loss of their viability. Indeed, we observed a two-fold decrease in the infection forming units of AS9 stocks when the bacteria were preincubated for about 40 min at 37°C in complete medium before being added to the cells. In comparison, it took about 120 min of preincubation for the wild type bacteria to observe a similar loss of infectivity (Figure 6C). This suggests that the viability of the bacteria that do not express CT622 is reduced.

## Discussion

Our results converge to identify CT622 as a secreted protein that plays multiple and crucial roles in the initiation and support of the *C. trachomatis* growth cycle.

We first demonstrated that CT622 translocation occurs through a T3S-dependent mechanism. Gong et al. also came to this conclusion but based on the use of a drug that was later shown not to be a specific inhibitor of T3S, therefore our report clarifies this uncertainty (Gong et al., 2011; Engström et al., 2013). Orthologs to CT622 in *C. pneumoniae* and *C. caviae* also appear to have a functional T3S signal and are only found throughout *Chlamydiaceae*, but not within the environmentally-adapted *Chlamydiales*, suggesting a potential functional role exclusive to interactions with higher order eukaryotes.

T3S effectors are commonly bound by a chaperone in the bacterial cytoplasm, prior to secretion. To date Slc1 and Mcsc are the only two chaperones for chlamydial T3S effectors identified, with CT584 being a third promising candidate (Pais et al., 2013). An unbiased pull-down experiment identified CT635 as a potential chaperone for CT622 and, unlike Slc1 and Mcsc, the CT635 primary sequence and predicted structure did not point to a potential chaperone activity. Direct pull-down experiments also revealed that Slc1 can interact with CT622, but to a lesser extent than CT635, and Mcsc did not pull-down the chlamydial effector. The interaction with CT635 required the first 94 amino acids of CT622 to be present, consistent with canonical T3S chaperones (Parsot et al., 2003). These properties, together with its small size and its ability to form dimers (unpublished data) suggest that CT635 may be a novel T3S chaperone, although this remains to be confirmed by further investigation. Interestingly CT622 and CT635 were detected in equimolar concentration in purified EB (Saka et al., 2011) and were quite abundant in the infectious form of the bacteria, similarly to what was observed for prepacked early effectors (Cossé et al., 2016). This observation suggests that CT622 might be secreted rapidly upon infection. However, unlike TarP or TepP, we did not detect any phosphorylation in CT622 upon translocation in the cytoplasm, precluding a kinetics analysis of its translocation.

Like the gene coding for the early effector TarP (Lutter et al., 2010), *ct622* shows a high density of single nucleotide polymorphism, both in ocular and genital strains (Kari et al., 2008; Harris et al., 2012). SNPs are concentrated in the N-terminal part (T3S signal and CT635 binding site) and in the linker region between the N-terminal and C-terminal domains (Figure S2), indicating that the polymorphism highlights CT622 domains that can undergo variations, maybe to escape immune recognition, without loss of function. Consistent with the hypothesis that CT622 is a potent target of the immune reaction to infection, a study involving 40 mice intravaginally infected with *C. muridarum* reported that antibodies against CT622 were detected in 85% of the mice (Zeng et al., 2012).

We determined the structure of the carboxy-terminal domain of CT622. Half of this domain showed some similarity with members of the polyprenyl transferase family. However, CT622 does not appear to catalyze geranylgeranylation, or to interact with prenyl groups. Thus, while the structural similarity between CT622 and GG transferases/synthases remains intriguing, further investigations will be needed to determine if it reflects a functional parenthood with this family of enzymes.

We obtained a deletion mutant of *ct622*, designated as AS9 (*ctl0886::aadA*), by using group II intron insertion (Johnson and Fisher, 2013). The ability to generate this strain demonstrates that CT622 is not absolutely required for bacterial development. There was however, a strong growth defect associated with this strain (e.g., 25-fold reduction in progeny formation). Bacteria were internalized less efficiently and host protein phosphorylation events that are normally triggered upon invasion were strongly reduced. Only a minority of the internalized bacteria developed into mature inclusions. Individual bacteria (as opposed to those located in an inclusion) observed at the ultrastructural level mostly displayed a morphology typical of EBs, suggesting that conversion to the RB stage was impaired, at least in a fraction of the AS9 population. It is possible that CT622 is not directly needed for the EB to RB transition, and that the loss of infectivity is due to defective EB formation or loss of EB viability in the absence of this effector protein.

While in the absence of CT622 most bacteria failed to develop, some gave rise to normal-looking inclusions. These AS9 inclusions were about two-fold smaller than wild type inclusions, and bacterial load was reduced by 50%, indicating that CT622, beyond its direct or indirect implication in the very early steps of infection discussed above, is also involved in sustaining RB proliferation. Consistent with this hypothesis, *ct622* mRNA were detected as early as 2 hpi (Gong et al., 2011), and we confirmed in the present study that CT622 accumulated to detectable levels in the host cytoplasm.

The functional study of effector proteins has benefited tremendously from the recent ability to perform targeted and non-targeted gene disruption in the *C. trachomatis* genome. CT622 is the first chlamydial effector identified to date whose genetic disruption has such a strong impact on bacterial development in cell culture, without being fully lethal. Absence of CT622 affects several steps of the infectious cycle, suggesting that CT622 is a multi-functional effector. This would not be surprising, as the bacteria rely on a limited number of effectors to modify the cell in multiple ways. However, some of the defects of the AS9 strains are possibly not directly due to a missing CT622-driven action, but might result indirectly from defective EB formation. Interestingly, absence of CPAF was reported to modify the abundance and processing of three T3S effectors, indicating that genetic invalidation of *cpaf* might also have indirect effects, by interfering with the function of T3S effectors (Patton et al., 2016). Future work addressing the function of CT622, as with other chlamydial secreted proteins, will need to consider this possible pitfall in the analysis of mutant strains.

## Materials and methods

### Cells and bacteria

HeLa cells (ATCC) were cultured in Dulbecco's modified Eagle's medium with Glutamax (DMEM, Invitrogen), supplemented with 10% (v/v) fetal bovine serum (FBS). Cells were routinely checked for absence of mycoplasma contamination by PCR. *C. trachomatis* LGV serovar L2 strain 434 (ATCC) were purified on density gradients as previously described (Scidmore, 2005). The *ipaB* and *mxiD* strains are derivates of M90T, the virulent wild-type strain of *S. flexneri*, in which the respective genes (*ipaB* and *mxiD*) have been inactivated (Allaoui et al., 1993). The *E. coli* strain *DH5*α was used for cloning purposes and plasmid amplification. Both *S. flexneri* and *E. coli* strains were grown in Luria-Bertani medium with ampicillin at 0.1 mg/ml.

### Immunofluorescence

HeLa cells grown on coverslips were infected with *C. trachomatis* LGV serovar L2 strain 434 with a multiplicity of infection (MOI) < 1 (unless specified differently) and fixed in 4% paraformaldehyde (PFA) (w/v) in PBS for 20 min at room temperature, except when staining the inclusion membrane with antibodies against CT813, which required 2% PFA. Cells were blocked and permeabilized in 0.3% Triton X-100 (v/v) in PBS for 5 min followed with 5 min incubation with 1% bovine serum albumin (BSA) in PBS. Antibodies were diluted in 1% BSA (w/v) in PBS. Mouse anti-MOMP (#11–114, IgG2b) and anti-flagM2 antibodies were from Argene and Sigma, respectively. Rabbit anti-CT621 and anti-Cap1 antibodies were described elsewhere (Muschiol et al., 2011; Gehre et al., 2016); mouse anti-CT813 was from D. G Zhong (University of Texas). Mouse Goat secondary antibodies were conjugated to Alexa488 (Molecular Probes), to Cy3 or Cy5 (Amersham Biosciences). Hoechst 33342 was from Molecular Probes. Images were acquired on an Axio observer Z1 microscope equipped with an ApoTome module (Zeiss, Germany) and a 63× Apochromat lens. Images were taken with an ORCA-flash4.OLT camera (Hamamatsu, Japan) using the software Zen.

### Plasmids and transfections

Genomic DNA from *C. trachomatis* D/UW-3/CX, *C. caviae* GPIC and *C. pneumoniae* TW183 was prepared from bacteria using the RapidPrep Micro Genomic DNA isolation kit (Amersham Pharmacia Biotech). The first 26–29 codons of *ct622*, and its homologs *cca00015* and *cpn0728*, including about 20 nucleotides upstream from the translation start sites, were amplified by PCR using primers listed in Table S5 and cloned into the pUC19cya vector as described (Subtil et al., 2001). Full length CT635 was cloned in the vector pKJ3 (Parsot et al., 2005) downstream of LacI operator binding sites, using BamHI and KpnI restriction sites, in frame with a N-terminal 6-Histidine tag. *ct622* (full length or trunctated of the first 94 codons) was cloned downstream of *ct635*, using KpnI and SalI restriction sites, for co-translation in an operon. attB-containing primers (Table S5, Gateway®, Life technologies) were used to amplify and clone *ct622* into a destination vector derived from the mammalian expression vector pCiNeo, providing an amino-terminal 3xflag tag, and into pDEST15 or pDEST17 (Gateway), for production of GST or HIS-tagged proteins, respectively. All constructs were verified by sequencing.

### Production of GST-CT622 protein and of rabbit polyclonal antibody to CT622

GST-CT622 and GST were expressed in BL21DE3(pLysS). Overnight cultures were inoculated into 500 ml LB broth containing 0.1 mg/mL ampicilin, incubated at 37°C until the optical density at 600 nm was 0.4–0.6. The production of recombinant protein was initiated with the addition of 0.25 mM isopropyl 1-thio-β-D-galactopyranoside (IPTG) and cultures were incubated at 37°C for 3 h. Cultures were then centrifuged at 4,000 × *g* for 20 min, and pellets were lysed for 30 min at 4°C in lysis buffer [0.3 M NaCl, 50 mM Tris-HCl pH 7.5 1% (v/v) Triton X-100 (Sigma), 5% (v/v) glycerol, 2 mM DTT], supplemented with 0.5 mg/ml lysozyme (Sigma #L6876), 330 U DNase I (Roche, #04716728001), and protease inhibitors cocktail (CIP, Sigma # P8340, 1:100). Insoluble material was removed by centrifugation at 17,000 × *g* for 20 min at 4°C. Supernatants were rotated on a rocking platform for 1 h onto 300 μl glutathione sepharose 4B beads (GE Healthcare) and transferred to columns. Beads were washed two times with the lysis buffer and then once with the following buffer: 50 mM Tris-HCl pH 7.5, 0.3 M NaCl, 5% glycerol, 2 mM DTT. GST fusion proteins on glutathione agarose beads were eluted in elution buffer (20 mM glutathione, 50 mM Tris-HCl pH 7.4, 300 mM NaCl, 5% glycerol), dialyzed overnight against 0.15 M NaCl, 30 mM Tris-Hcl pH 7.5, 5% glycerol, 1 mM DTT and stored at −80°C. An additional step of purification by gel filtration on HiLoad columns 16/60 Superdex-200 was added before using the proteins in MST experiments.

GST-CT622 was used as immunogen for the production of specific polyclonal antibodies in New Zealand White rabbits (produced by Agro-Bio, La Ferté Saint-Aubin, France). HIS-CT622 was expressed in *E. coli* and purified on Ni^2+^-NTA-Sepharose column as described below. Sera were purified against HIS-CT622.

### *Shigella* heterologous secretion assay

Analysis of secreted proteins was performed as described previously (Subtil et al., 2001). Briefly, 1 ml of a 30°C overnight culture of *S. flexneri ipaB* or *mxiD* transformed with different Cya chimeras was inoculated in 30 ml of LB broth with 0.1 mg/ml ampicillin and incubated at 37°C for 4 h. For experiments using full-length proteins expression was induced by adding 150 μM IPTG over the 4 h growth period. Bacteria were then harvested by centrifugation and the supernatant was filtered through a Millipore filter (0.2 μm). To precipitate the proteins 1/10 (v/v) of trichloroacetic acid was added to the supernatant and the precipitate as well as the bacterial pellet resuspended in sample buffer for analysis by SDS-PAGE and immunoblot.

### Immunodetection and pull-down experiments

#### Immunodetections

Proteins were subjected to SDS-PAGE, transferred to Immobilon-P (PVDF) membranes and immunoblotted with the proper primary antibodies diluted in 1X PBS containing 5% milk and 0.01% Tween-20. Primary antibodies used were rabbit anti-RNA polymerase (Abcam, clone 8RB13), mouse anti-actin (Sigma #5441 clone AC-15), mouse anti phospho-tyrosine clone 4G10 (ascite generated by the laboratory), rabbit anti-TarP (generously given by Dr. Ted Hackstadt), rabbit anti-DnaJ (Stressgen SPA-410), mouse anti-cya, rabbit anti-CRP, and rabbit anti-IpaD antibodies generously given by Drs. N. Guiso, A. Ullmann and C. Parsot, respectively (Institut Pasteur, Paris). Goat anti-mouse and anti-rabbit IgG-HRP (GE Healthcare) were used at 1:10,000 dilution. Blots were developed using the Western Lightning Chemiluminescence Reagent (GE Healthcare).

#### Pull-downs

For GST pulldowns, cells were lysed in 0.5–0.05% NP40 lysis buffer described above. EBs were lysed in the same buffer and were additionally sonicated for 15 min (30/30 s cycles, power High) with Bioruptor (Diagenode). Lysates were centrifuged at 17,000 × *g* for 15 min at 4°C and precleaned with glutathione-agarose beads with 20 μg of GST for 90 min at 4°C in a rocking platform. Equal amount of precleaned supernatants were incubated with 50 μl of a 50% slurry of glutathione sepharose 4B beads and 30 μg of purified GST or GST-CT622 for 90 min at 4°C, on a rocking platform. After a brief centrifugation, beads were washed five times with cold GST lysis buffer. The bound proteins were eluted with urea buffer (8M urea, 1% SDS, 150 mM NaCl, 30 mM Tris-HCl pH 8.0) then identified by mass spectrometry by the Proteopole of the Institut Pasteur.

For HIS-CT635 pull-down, an overnight culture of *E. coli* transformed with a plasmid coding for HIS-CT635 and CT622, CT622 alone, or HIS-CT635 and Δ94CT622 was diluted 1:25 in 50 ml of fresh LB supplemented with 0.1 mg/ml ampicillin. Culture were grown to an OD_600_ of ~0.5 and protein expression was induced with 0.1 mM IPTG. Three h later, bacteria were collected, resuspended in 3.5 ml of lysis buffer (300 mM NaCl, 50 mM NaH_2_PO_4_, pH 8.0) supplemented with 5 mM imidazole, and lysed by sonication. Soluble material was incubated for 1 h at 4°C with 0.3 ml of Ni^2+^-NTA-Sepharose slurry previously washed three times with lysis buffer, and transferred to a column. The beads were washed with 2 × 10 ml of lysis buffer supplemented with 10 and 50 mM imidazole, respectively. Proteins were eluted by increasing the imidazole concentration to 250 and 500 mM. The fractions eluted in 500 mM imidazole are shown.

### Construction of pTTmut9*aadA*

The intron in pDFTT3*bla* was retargeted for *ctl0886* using primer sequences listed in Table S5 to create an insertion after nucleotide 447 of the *ctl0886* coding sequence, as described in TargeTron^TM^ (Sigma). The finale PCR product was cloned between the HindIII and BsrGI restriction sites of pDFTT3, creating pTTmut9*bla*. The *aadA* gene was digested from pDFTT3*aadA* (Lowden et al., 2015) using MluI, purified on a 0.8% agarose (w/v) gel, and ligated into a similarly digested pTTmut9*aadA*. The ligation product was transformed into *E. coli* DH5α and transformants were selected on LB agar supplemented with 100 μg/ml of spectinomycin at 30°C. Transformants were tested for the orientation of *aadA* using colony PCR with the primers GIIR and aadA5 and 2X PCR Master Mix (Thermo Fisher). A PCR-positive colony was selected for plasmid extraction (GeneJet Plasmid Miniprep Kit, Thermo Fisher) following overnight growth in LB supplemented with chloramphenicol (20 μg/ml) and spectinomycin (100 μg/ml).

### Isolation of AS9—*C. trachomatis* L2 *ctl0886*::GII(*aadA*)

AS9 was generated using the methods described in Lowden et al. for the *aadA*-based vectors. The L2 strain used was L2 434/Bu ACE051 originating from Tony Maurelli's lab (University of Florida). After five passages in L2 cells under spectinomycin selection (500 μg/ml), EBs were harvested and clonal isolates were obtained using the plaque assay (L2 cells, 14 days for growth with spectinomycin). Nine plaques were picked and immediately used to infect L2 cells in a 96 well plate (EB attachment mediated via centrifugation) with selection. After 48 h, the wells were scraped and the contents were used to infect L2 cells in a 24 well plate with selection. Inclusions were visible at 48 h for wells containing plaques 1, 2, 5, 6, 7, and 9 (designated p1, p2, p5, p6, p7, and p9). The contents of the wells were collected and used to infect L2 cells in T25 flasks. After 48 h, the cells were harvested, lysed via sonication, and the EBs were pelleted by centrifugation at 13,000 × *g*, 4°C, 15 min. The EBs were suspended in 2 ml of SPG and stored at −80°C. Stocks were tittered using the inclusion forming unit assay.

### Analysis of AS9 plaque isolates

Genomic DNA was extracted from each clone using the DNeasy Blood and Tissue kit from Qiagen. PCR was performed using 2X PCR Master Mix and products were analyzed on 1% agarose gels (see Table S5 for primers). The hyp08F/R primers were used for detection of the L2 plasmid, GIInewF/R for detection of the GII intron and 886seqF/R for amplifying *ctl0886*. Gels were stained with ethidium bromide and viewed under UV trans-illumination. To sequence the insertion locus, the PCR product generated from the 886seqF/R PCR reaction was excised from the gel, purified (GeneJet Gel extraction kit, Thermo Fisher), and ligated into pJET (Thermo Fisher). Ligation products were transformed into DH5α and transformants were selected on LB agar supplemented with spectinomycin (100 μg/ml). A colony was then grown overnight in LB supplemented with ampicillin (100 μg/ml) and spectinomycin (100 μg/ml) for plasmid extraction. The insert was sequenced via Sanger sequencing (Macrogen USA) using the primers pJETF and pJETR.

For protein analysis, EBs were mixed with Laemmli plus β-mercaptoethanol and heated at 95°C for 5 min. Samples were then run on 12% SDS-PAGE gels and stained with Coomassie Brilliant Blue for total protein analysis or transferred to nitrocellulose for anti-MOMP western blot analysis. For anti-CTL0886 western blots, 8% SDS-PAGE gels were used. Blots were blocked with 5% milk (w/v) TBS (mTBS) following transfer and incubated with either mouse anti-MOMP antibodies (1:1,000, Abcam) or rabbit anti-CTL0886 antibodies (1:1,000) overnight at 4°C. Blots were then washed with 0.05% tween-20 TBS (TTBS) and probed for 1 h at room temperature with HRP-conjugated goat anti-mouse or anti-rabbit secondary antibodies diluted 1:1,000 in mTBS. Finally, blots were washed with TTBS followed by TBS, incubated with chemiluminescent substrate (Millipore), and imaged using a Bio-Rad Chemidoc MP with Image Lab software.

### Complementation of the AS9 strain

The *ctl0886* gene with its endogenous promoter, and a nucleotide sequence coding for a flag tag immediately upstream of the stop codon, was cloned into the pBOMB4 plasmid (Bauler and Hackstadt, 2014; see Table S4 for primers). The AS9 strain was stably transformed with this plasmid as described (Wang et al., 2011).

### Reinfection assays

Two AS9 clones, AS9p1 and AS9p2, were pooled, expanded, and the resulting progeny, designated as AS9, was purified on a density gradient as described (Scidmore, 2005), and the titer determined by infecting fresh HeLa cells with serial dilutions. To compare the fitness of the AS9 strain to that of the parental strain ACE051 HeLa cells grown in 24-well plates were infected in duplicates at different MOI ranging between 0.02 and 0.2. Forty-eight hours later, one set of cells was used for a titration assay, while one well was visually chosen (around 10% infection) to reinfect fresh HeLa cells. Cells in the selected well were scraped, collected with the supernatant, and vortexed for 10 s with 1 mm glass beads before spinning at 1,000 rpm for 5 min. Fresh HeLa cells were inoculated with serial dilutions of the supernatant, and 24 h later flow cytometry was used to determine the infection rates as described elsewhere (Vromman et al., 2014). Briefly, cells were washed with PBS and gently trypsinized. After a washing step in PBS cells were fixed in PFA 2% in PBS and bacteria were stained using anit-Hsp60 antibody (Affinity BioReagents, MA3-023) followed with Alexa488-conjugated secondary antibodies. Infection rates were measured using a Gallios flow cytometer (Beckton Coulter). The titration assay performed on the first round of infection allowed to correct for variations between experiments, and the data were expressed as inclusion forming units (IFU) obtained at 48 hpi for an input of 10∧3 IFU.

### Entry measurements and protein phosphorylation assay

To measure bacterial entry cells grown on coverslips in 24-well plates were incubated for 30 min with bacteria (AS9 or parental ACE051 strain) at 4°C to allow bacterial attachment and then transferred to 37°C for variable times. The cells were then fixed with 4% PFA, and extracellular bacteria were stained with anti-LPS antibodies (clone ACI, Progen 1:1,000) followed with Alexa647-conjugated antibodies. The cells were then permeabilized, and intracellular bacteria were stained with anti-LPS antibodies followed with Alexa488-conjugated antibodies. Images were taken randomly and analyzed with the ChlamEntry protocol of the ICY softaware as described (Vromman et al., 2014). To assay protein phosphorylation upon bacterial entry cells in 24-well plates were incubated with decreasing number (m.o.i = 20-10-5-2.5) of the wild-type, AS9 or AS9_complCT622_ bacteria for 40 min at 4°C before shifting the plates to 37°C. Samples were collected at 20 or 120 min by washing the cells once in PBS and adding 90 ul of Laemmli sample buffer. Proteins were resolved by SDS PAGE, transferred onto PVDF membranes and subjected to immunoblot. The top half of the membranes were first probed with anti-phosphotyrosine antibody (4G10) before gentle sCT622 and re-blot with anti-TarP antibody. The bottom half of the membrane was probed with anti-MOMP antibody.

### Prenylation reactions and prenyl binding

Prenylation reactions were performed using Rab Escort Protein 1 (Rep1) purified from Insect cells and RabGGTase α+β copurified from *E. coli* as previously described (Wu et al., 2010). Rab6 was purified as described for Rab8 but without ATP (Bleimling et al., 2009). Farnesyl-NBD-pyrophoshate was used as it was demonstrated to serve as a good fluorescent geranylgeranyl-pyrophosphate analog (Dursina et al., 2006). For each assay, 0.5 nmol RabGGTase, 2.5 nmol GST-Δ345CT622 (inhibition test), or 0.5 nmol GST-CT622 (prenylation test) were mixed with 0.5 nmol farnesyl-NBD, 0.375 nmol Rep1, and 0.25 nmol Rab6 (wild type). Prenylation reactions were performed over night at 20°C. Proteins were separated in 15% SDS-PAGE and fluorescence was observed using the Ethidium Bromide mode of a gel reader.

Fluorescence measurements were performed using a Cary Eclipse Fluorescence Spectrometer from Agilent Technologies. GGTaseI α+β from *E. coli* as previously described (Oesterlin et al., 2012). The buffer used was 50 mM Hepes pH 7.2, 50 mM NaCl, 5 mM DTE. NBD fluorescence was excited at 487 nm and emission was measured in scan mode from 515 to 630 nm. All measurements were performed in a volume of 1 ml with a cuvette with a magnetic stirrer.

### Statistical analyses

The R environment was used for all the analyses. Statistical significance was set to *P* ≤ 0.05. In each figure, type I error was controlled by correcting the p values according to the Benjamini & Hochberg method [“BH” option in the p.adjust() function of R]. The results are summarized in Table S5. Internalization data were analyzed using a linear model, to take into account potential interaction effects between the strains and the time lapses. Internalization of WT, AS9 strain, and AS9c_ompCT622_ bacteria were compared 2 by 2, for each time lapse, using the contrast() function of the contrast package. The same was applied to qPCR data.

### Mass spectrometry

#### In solution digestion of proteins

Proteins from the pull-down fraction were solubilized in denaturation buffer [2-amino-2-hydroxyméthyl-1,3-propanediol (Tris) 100 mM pH 8.0, 8M urea]. Reduction of disulfide bridges was performed in 5 mM dithiothréitol (DTT) for 30 min at room temperature; alkylation was performed in 20 mM iodocacetamide in the dark for 30 min at room temperature. Five hundred nanograms of LysC (Promega, MAdison, WI, USA) were added to each sample and digestion was performed at 30°C for 3 h. Samples were diluted in 100 mM ammonium bicarbonate (BA) until the urea concentration was below 1.5 M. Five hundred nanograms of trypsin (Promega, Madison, WI, USA) was added for each sample and digestion was performed at 37°C over-night. Digestion was stopped by adding 1% formic acid (FA). Resulting peptides were purified using the SPE C18 method. Briefly, the C18 phase (Sep-Pak, Waters) was activated in methanol, rinsed once in 80% acetonitrile (ACN) 0.1% FA, and washed thrice in 0.1% FA. Samples were loaded twice on the top of the cartridge. The resin was washed thrice in 0.1% FA and once in 2% ACN 0.1% FA. Peptides were eluted in 50% ACN 0.1% FA and concentrated to almost dryness in a speed vac.

#### Mass spectrometry analysis, database search, and protein identification

Digested peptides were analyzed by nano LC-MS/MS using an EASY-nLC 1000 (Thermo Fisher Scientific) coupled to a Q Exactive Orbitrap mass spectrometer. For input samples, about 1 μg of each sample (dissolved in 0.1% FA) were loaded and separated at 250 nl/min on a home-made C_18_ 50 cm capillary column picotip silica emitter tip (75 μm diameter filled with 1.9 μm Reprosil-Pur Basic C_18_-HD resin, (Dr. Maisch GmbH, Ammerbuch-Entringen, Germany)) equilibrated in solvent A (0.1% FA). The peptides were eluted using a two slopes gradient of solvent B (0.1% FA in ACN) from 2 to 5% in 5 min, 5 to 30% in 150 min, 30 to 60% in 70 min at 250 nL/min flow rate (total length of the chromatographic run was 240 min including high ACN level steps). For pull down samples, half of the sample was injected on a 30 cm long column and peptides were separated with a 120 min long gradient. The Q Exactive (Thermo Fisher Scientific, Bremen) was operated in data-dependent acquisition mode with the XCalibur software 2.2 (Thermo Fisher Scientific, Bremen). Survey scan MS were acquired in the Orbitrap on the 300–1,700 *m/z* range with the resolution set to a value of 70,000 at *m/z* = 400 in profile mode (AGC target at 1^E^6). The five most intense ions per survey scan were selected for HCD fragmentation (NCE 27), and the resulting fragments were analyzed in the Orbitrap at 17,500 of resolution (m/z 400). Isolation of parent ion was fixed at 1.6 m/z and underfill ratio at 1%. Dynamic exclusion was employed within 45 s. Data were searched using MaxQuant (1.4.1.2 version) (with the Andromeda search engine) against the Human database from SwissProt and TrEMBL (2014.01.14, 88,500 entries including 39,715 from swiss prot), and the *C. trachomatis* database from Uniprot (2014.10.20, 889 entries). The following search parameters were applied: carbamidomethylation of cysteines was set as a fixed modification, oxidation of methionine, and protein N-terminal acetylation were set as variable modifications. The mass tolerances in MS and MS/MS were set to 5 ppm for each, respectively. Maximum peptide charge was set to 7 and 5 amino acids were required as minimum peptide length. Peptides and proteins identified with an FDR lower than 0.1% were considered as valid identification. Label free analysis was done by using the “match between run” feature of MaxQuant (3 min time window). LFQ data were used to performed statistical analysis between conditions of infection.

### Cloning, overexpression, and purification of CTL0886

The following gene fragments were amplified from *C. trachomatis* (serovar L2 434/Bu) genomic DNA via PCR and subcloned into *BamH*I/*Not*I-digested pT7HmT (Geisbrecht et al., 2006): residues 94-651 (CTL0886^FL^), 94-322 (CTL0886^N^), and 353-651 (CTL0886^C^). Expression and purification were performed for all 3 constructs in a similar manner as previously published (Barta et al., 2015). The purified protein was concentrated to 10 mg/ml and buffer exchanged by ultrafiltration into 10 mM Tris-HCl (pH 7.5), 50 mM NaCl, and stored at 4°C for further use. Selenomethionine (SeMet)-substituted CTL0886^C^ was grown according to standard protocols (Doublié, 2007), purified as described above (all buffers contained 5 mm β-mercaptoethanol), and concentrated to 10 mg/ml in 10 mM Tris-HCl (pH 7.5), 50 mM NaCl buffer for crystallization.

### Reductive methylation

CTL0886^N^ and CTL0886^C^ failed to produce diffraction quality crystals despite extensive screening. Reductive methylation of surface exposed lysines was performed in order to enhance potential intra- and inter-molecular interactions involving these residues (Walter et al., 2006). Each purified protein was dialyzed into PBS at a concentration of 10 mg/ml and pipetted into a 5 ml eppendorf tube covered in foil. Fresh solutions of DMAB (1 M) and formaldehyde (1 M) were prepared. For each 1 ml of protein, 20 ul of DMAB and 40 ul of formaldehyde were added to the protein solution, which was then gently rocked in the dark at 4°C for 2 h. This step was repeated two more times. Finally, 10 ul of DMAB per 1 ml of protein was added, followed by gentle rocking overnight in the dark at 4°C. The reaction was quenched and excess reagent was removed by a final size exclusion purification step [20 mM Tris-HCl (pH 8.0), 200 mM NaCl]. Methylated proteins were concentrated to 10 mg/ml and buffer exchanged by ultrafiltration into 10 mM Tris-HCl (pH 7.5), 50 mM NaCl, and stored at 4°C for further use. SeMet-substituted CTL0886^C^ was methylated in an equivalent manner.

### Crystallization

Methylated *C. trachomatis* CTL0886^N^ and CTL0886^C^ were crystallized by vapor diffusion in Compact Jr. (Emerald Biosystems) sitting drop plates at 20°C. Specifically, 0.5 μl of protein solution [10 mg/ml in 10 mM Tris-HCl (pH 7.5) and 50 mM NaCl] was mixed with 0.5 μl of reservoir solution. Crystals of CTL0886^N^ were produced with a reservoir solution containing 30% (w/v) Polyethylene glycol 1,500, from the Crystal HT screen condition D7 (Hampton). Single bipyramidal-shaped crystals appeared after 2 days and reached a maximum size of ~100 microns after 5 days. Crystals of CTL0886^C^ were produced with an optimized reservoir solution containing 0.1 M Bis-Tris (pH 7.2), 1.9 M ammonium sulfate, from the Index HT screen condition A4 (Hampton). Large block-shaped crystals appeared after 1 day and reached a maximum size of ~300 microns after 3 days. Crystals were flash-cooled in a cryoprotectant solution consisting of 40% (w/v) Polyethylene glycol 1,500 or saturated ammonium sulfate for CTL0886^N^ and CTL0886^C^, respectively.

Methylated SeMet-CTL0886^C^ crystals were obtained in essentially the same manner as described above, and large block-shaped crystals were harvested as described above.

### Diffraction data collection, structure determination, refinement, and analysis

X-ray diffraction data were collected at 1.000 Å at 100 K using a Dectris Pilatus 6M pixel array detector at beamline 17ID at the APS IMCA-CAT (Table S2). Crystals of CTL0886^N^ diffracted at best to 6.3 Å and will not be discussed any further within this manuscript. Following data collection, individual reflections were integrated with XDS (Kabsch, 2010). Laue class analysis and data scaling were performed with Aimless (Evans, 2011), which suggested the Laue class was *m*-3 with a likely space group of *I*23 or *I*2_1_3.

Experimental phase information was obtained for the CTL0886^C^ structure by Se-SAD using AutoSol within the Phenix suite (Adams et al., 2002, 2010), which identified nine unique selenium atoms in the asymmetric unit. Phenix Autobuild correctly traced 148/299 Cα atoms (Map-model CC = 0.58, *R*_work_/*R*_free_ = 41.32/46.48) within a single CTL0886^C^ polypeptide. Anomalous phases were combined with the complete 1.90 Å native diffraction dataset using CAD (Collaborative Computational Project, 1994). Subsequently, Phenix.Autobuild was then used to trace 241/299 of the expected amino acids from the combined experimental maps with *R*_work_/*R*_free_ = 23.05/26.88. Structure refinement was carried out using Phenix. One round of individual coordinates and isotropic atomic displacement factor refinement was conducted, and the refined model was used to calculate both 2*F*_*o*_*-F*_*c*_ and *F*_*o*_*-F*_*c*_ difference maps. These maps were used to iteratively improve the model by manual building with Coot (Emsley and Cowtan, 2004; Emsley et al., 2010) followed by subsequent refinement cycles. TLS refinement (Painter and Merritt, 2006) was incorporated in the final stages to model anisotropic atomic displacement parameters. Ordered solvent molecules were added according to the default criteria of Phenix and inspected manually using Coot prior to model completion (*R*_work_/*R*_free_ = 21.09/25.79). Additional information and refinement statistics are presented in Table S5. Regions of poor map quality precluded the modeling of the following residues, chain A: 428–429, 523–530, 590–597, and 651.

### Multiple sequence alignments and figure modeling

Representations of all structures were generated using PyMol (www.pymol.org). Multiple sequence alignments were carried out using ClustalW (Thompson et al., 1994) and aligned with secondary structure elements using ESPRIPT (Gouet et al., 1999). Three-dimensional structures were superimposed using the Local-Global Alignment method (LGA) (Zemla, 2003).

### Electron microscopy

Cells were fixed with 2.5% glutaraldehyde (v/v) (electron microscopy sciences) in 0.1 M cacodylate buffer, pH 7.4, for 1 h at room temperature. After several washes in cacodylate they were post-fixed with 1% osmium tetroxide (w/v) in cacodylate for 1 h at RT. After several washes with water the cells were progressively dehydrated with increasing concentrations of ethanol from 25 to 100%. The cells were then gradually embedded in epoxy resin. After overnight polymerization at 60°C, 50–70 nm thin sections were cut in an ultra-microtome (Ultracut, Leica) and cells imaged after post-staining with uranyl acetate and lead citrate in a T12-FEI transmission EM operated at 120 kV.

### Quantitative reverse transcription PCR

Total RNA was isolated from 5 × 10^5^ HeLa cells infected with wild type or AS9 bacteria after 1, 3, or 6 h of infection (MOI of 10) with the RNeasy Mini Kit with DNase treatment (Qiagen) according to the manufacturer's protocol. For the 0 h time point bacteria were added directly to the cell pellet before proceeding to RNA extraction. RNA concentrations were determined by NanoDrop and 500 ng RNA were treated with DNAse (Roche) in the presence of RNAsine (Promega). Reverse transcription (RT) was performed with random hexamer primers (Roche) and M-MLV reverse transcriptase (Promega). cDNA in the samples was determined by quantitative PCR (qPCR) using chlamydial primers with LightCycler 480 system using FastStart Universal SYBR Green Master (Roche). Genomic DNA (gDNA) was purified for each time point from wells infected in parallel with the DNeasy Blood and Tissue Kit (Qiagen), and the gDNA in the samples determined by qPCR using chlamydial primers. Histograms depicts the amount of cDNA normalized to bacterial gDNA content at each time point, to account for differences in kinetics of entry between the two strains. Primers are listed in Table S4, their specificity was ensured through the analysis of melting curves.

## Author contributions

MC, MB, DF, LO, PH, and AS: designed experiments and interpreted data; MC: performed most of the experiments; MB and PH: performed the structural analysis; DF: obtained the AS9 strain; LO: performed the prenylation and prenyl binding assays; SP: performed EM experiments; BN and SP: provided technical assistance; GM: performed statistical analyses; MC, MB, DF, and AS: wrote the manuscript.

### Conflict of interest statement

The authors declare that the research was conducted in the absence of any commercial or financial relationships that could be construed as a potential conflict of interest.

## Supplementary material

Table S1 Mass spectrometry identification of CT622 partners in bacterial lysates. Pull-downs were performed in duplicates on *C. trachomatis* L2 lysates using GST-CT622 or GST alone as baits as described in the Materials and Methods section. The proteins identified by mass spectrometry in the pull-down fractions are reported in the table, with the number of peptides identified for each protein and the % of the protein they covered (in bold when the number of peptides was significantly higher in the pull-down performed with GST-CT622 compared to the control with GST). The experiment was performed twice independently. Only MOMP and CT635 were identified in the two experiments.

Table S2 X-ray diffraction data and refinement statistics.

Table S3 CT622^C^ DALI search statistics.

Table S4 Primers used.

Table S5 Statistical analyses.

Figure S1 Detection of CT622 with rabbit polyclonal antibody. **(A)** HeLa cells were infected with LGV L2 at MOI = 0.5. At the indicated time, cells were collected and total cell lysate were prepared in urea buffer. Proteins were resolved in 8% polyacrylamide gels and probed by immunoblotting with antibodies to CT622, bacterial RNA polymerase, or actin. The expected molecular weight of CT622 is 68 kDa (asterisk). A cross-reaction product around 78 kDa was also detected by anti-CT622 antibodies. NI, not infected. **(B)** HeLa cells were infected with LGV L2 for 24 h before fixation and permeabilization. Purified anti-CT622 antibodies (0.5 μg/ml) were preincubated or not for 30 min with an excess (10 μg/ml) of the indicated recombinant protein before contact with the coverslip. Bacteria were labeled with mouse anti-MOMP antibodies. Secondary antibodies used were Alexa488-conjugated anti-rabbit antibodies (green) and Cy5-conjugated anti-mouse antibodies (red). DNA was stained with Hoechst (blue in merge picture). Most of the anti-CT622 signal disappeared in the presence of an excess of GST-CT622 and not of an irrelevant protein. A faint signal remained, likely corresponding to the bacterial cross-reaction product observed by western blot. Scale bar = 10 μm.

Figure S2 Multiple Sequence Alignment of CT622 and Orthologs from *Chlamydiaceae*. Sequence alignment was generated using ClustalW and rendered with ESPRIPT. The numbers above the sequences correspond to *C. trachomatis* CTL0886. The secondary structure of CTL0886^C^ is shown above the alignment. Residues are colored according to conservation (*cyan*, identical, and *purple*, similar) as judged by the BLOSUM62 matrix. Red bar highlights stretch of negatively charged side chains.

Figure S3 Purification of Recombinant CTL0886. Purified CTL0886^FL^ (residues 94–651) as well as a degraded full-length sample were analyzed by SDS-PAGE. Two arrows indicate the presence of overlapping protein bands in the degraded sample. LC-MS/MS analysis indicated they contained residues 94–334 (dashed arrow) and 353–651 (solid arrow). These fragments were subsequently expressed and purified, CTL0886^N^ (residues 94–322) and CT0886^C^ (residues 353–651).

Figure S4 Functional assays on CT622 activity regarding prenylation reactions. **(A)** Fluorescence scan to reveal farnesyl-NBD fluorescence on an SDS-PAGE of protein samples after prenylation tests. In the left two lanes it was tested whether GST-CT622 can prenylate Rab6 in the presence of Rab Escort Protein. GST-CT622 (only CT622) does not prenylate Rab6 whereas the positive control with RabGGTase (only GGT) shows a clear band with prenylated protein. 0.5 nmol of RabGGTase (GGT) or GST-CT622 (CT622) were used. In the right two lanes 2.5 nmol GST-Δ345CT622 was added to a prenylation reaction with 0.5 nmol RabGGTase to test whether CT622 can inhibit prenylation by RabGGTase by competitive binding (GGT+-Δ345CT622). No inhibition was observed. **(B)** Fluorescence spectrometry measurements of a titration to test binding of farnesyl-NBD-pyrophosphate (500 nM) to GST-Δ345CT622. No fluorescence change can be observed upon addition of GST-Δ345CT622. As a final step 200 nM GGTaseI was added to show fluorescence increase upon nucleotide binding. **(C)** Positive control: exemplary fluorescence spectrometry measurements of a titration with GGTaseI α+β to show farnesyl-NBD fluorescence change (650 nM) upon protein binding.
