# Supplementary material for: The Loss of Expression of a Single Type 3 Effector (CT622) Strongly Reduces Chlamydia trachomatis Infectivity and Growth
Source: Front Cell Infect Microbiol. 2018 May 15;8:145. doi: 10.3389/fcimb.2018.00145 (PMC5962693; doi:10.3389/fcimb.2018.00145)
Supplement: Table S1 — Mass spectrometry identification of CT622 partners in bacterial lysates. Pull-downs were performed in duplicates on C. trachomatis L2 lysates using GST-CT622 or GST alone as baits as described in the Materials and Methods section. The proteins identified by mass spectrometry in the pull-down fractions are reported in the table, with the number of peptides identified for each protein and the % of the protein they covered (in bold when the number of peptides was significantly higher in the pull-down performed with GST-CT622 compared to the control with GST). The experiment was performed twice independently. Only MOMP and CT635 were identified in the two experiments. [file Table_1.PDF]

Table S1

	Protein used in pull down		GST-CT622 (1)		GST-CT622 (2)		GST (1)		GST (2)	
Experiment 1	Gene names	Protein ID	Unique peptides	Sequence coverage [%]	Unique peptides	Sequence coverage [%]	Unique peptides	Sequence coverage [%]	Unique peptides	Sequence coverage [%]
	ctl0003 (ct635)	tr|B0B8L0|	**4**	20,8	**4**	20,8	1	8,3	1	8,3
	ctl0623	tr|B0B8J8|	**1**	4,2	0	0	0	0	0	0
	momp	sp|P06597|	**2**	10,4	**2**	10,4	0	0	0	0

	Protein used in pull down		GST-CT622 (1)		GST-CT622 (2)		GST (1)		GST (2)	
Experiment 2	Gene names	Protein ID	Unique peptides	Sequence coverage [%]	Unique peptides	Sequence coverage [%]	Unique peptides	Sequence coverage [%]	Unique peptides	Sequence coverage [%]
	ahpC	tr|B0B8H7|	0	0	1	9,7	0	0	0	0
	copD	tr|B0B8F3|	**1**	**6,6**	2	10	0	0	0	0
	ctl0003 (ct635)	tr|B0B8L0|	**1**	8,3	**3**	22,2	0	0	0	0
	ctl0286	tr|B0B9D8|	2	22	1	8	2	22	1	8
	dnaK	sp|B0B7W6|	**1**	1,4	**2**	2,6	0	0	0	0
	EFTU	sp|B0B7N8|	**1**	2,3	**1**	2,3	0	0	0	0
	fliY	tr|B0B859|	**2**	16,5	**2**	11,1	0	0	0	0
	groL	sp|B0B9L8|	0	0	1	2,2	1	2,2	0	0
	momp	sp|P06597|	**11**	46,4	**8**	29,2	4	15,5	2	4,8
	omcB	sp|B0B815|	9	23,8	8	20,7	7	19,6	7	17,6
	pmpC	tr|B0B7Y4|	0	0	1	0,9	0	0	0	0
