# Supplementary material for: The Loss of Expression of a Single Type 3 Effector (CT622) Strongly Reduces Chlamydia trachomatis Infectivity and Growth
Source: Front Cell Infect Microbiol. 2018 May 15;8:145. doi: 10.3389/fcimb.2018.00145 (PMC5962693; doi:10.3389/fcimb.2018.00145)
Supplement: Table S2 — X-ray diffraction data and refinement statistics. [file Table_2.PDF]

Table S2. X-ray Diffraction Data and Refinement Statistics

	CT622[C] Native	CT622[C] SeMet
**PDB ID**	5UE0	
**Data Collection**		
Unit-cell parameters (Å)	*a*=121.810	*a*=121.124
Space group	*I*23	*I*23
Resolution (Å)[1]	60.91 – 1.90 (1.94 – 1.90)	85.65 – 2.95 (3.19 – 2.95)
Wavelength (Å)	1.0000	0.97937
Temperature (K)	100	100
Observed reflections	235,316 (13,952)	123,406 (26,052)
Unique reflections	23,829 (1,516)	6,372 (1,304)
$<I/\sigma(I)>$[1]	22.6 (2.6)	18.6 (2.4)
Completeness (%)[1]	100.0 (100.0)	100.0 (100.0)
Multiplicity[1]	9.9 (9.2)	19.4 (20.0)
R_{merge} (%)[1, 2]	5.5 (102.2)	12.4 (124.2)
R_{meas} (%)[1, 4]	6.1 (115.4)	13.0 (130.7)
R_{pim} (%)[1, 4]	2.7 (52.8)	4.0 (40.4)
$CC_{1/2}$ [5]	99.9 (77.9)	99.9 (82.5)
**Phasing**		
Anom. Completeness (%)[1]		100.0 (100.0)
Anom. Multiplicity[1]		9.8 (9.8)
FOM		0.4
**Refinement**		
Resolution (Å)	32.56 – 1.90 (1.99 – 1.90)	
Reflections (working/test)	23,796/1,135 (2,801/140)	
R_{factor} / R_{free} (%)[3]	21.09/25.79 (39.28/41.31)	
No. of atoms (Protein/Ligand/Water)	4,091/5/98	
**Model Quality**		
R.m.s deviations		
Bond lengths (Å)	0.012	
Bond angles (°)	1.065	
Average *B*-factor (Å^2)		
All Atoms	50.9	
Protein	50.7	
Solvent	55.9	
Coordinate error, maximum likelihood (Å)	0.29	
Ramachandran Plot		

Most favored (%)	98.85
Additionally allowed (%)	1.15
Outliers (%)	0.00

[1]Values in parenthesis are for the highest resolution shell.

[2]$R_{\mathrm{merge}} = \Sigma_{hkl}\Sigma_i\, |I_i(hkl) - \langle I(hkl)\rangle| \,/\, \Sigma_{hkl}\Sigma_i\, I_i(hkl)$, where $I_i(hkl)$ is the intensity measured for the ith reflection and $\langle I(hkl)\rangle$ is the average intensity of all reflections with indices hkl.

[3]$R_{\mathrm{factor}} = \Sigma_{hkl}\, ||F_{\mathrm{obs}}\ (hkl)| - |F_{\mathrm{calc}}\ (hkl)|| \,/\, \Sigma_{hkl}\, |F_{\mathrm{obs}}\ (hkl)|$; R_{free} is calculated in an identical manner using 5% of randomly selected reflections that were not included in the refinement.

[4]R_{meas} = redundancy-independent (multiplicity-weighted) R_{merge}{Evans, 2011 #249;Evans, 2006 #17}. R_{pim} = precision-indicating (multiplicity-weighted) R_{merge}{Diederichs, 1997 #45;Weiss, 2001 #167}.

[5]$CC_{1/2}$ is the correlation coefficient of the mean intensities between two random half-sets of data {Karplus, 2012 #303;Evans, 2012 #293}.
