# Supplementary material for: The Loss of Expression of a Single Type 3 Effector (CT622) Strongly Reduces Chlamydia trachomatis Infectivity and Growth
Source: Front Cell Infect Microbiol. 2018 May 15;8:145. doi: 10.3389/fcimb.2018.00145 (PMC5962693; doi:10.3389/fcimb.2018.00145)
Supplement: Table S3 — CT622C DALI search statistics. [file Table_3.PDF]

Table S3. CT622^C DALI Search Statistics

CT622^C Domain 1 Structural Homology					
Top 3 (unique) DALI scores					
**Protein name**	**PDB code**	**Z-score[a]**	**RMSD**	**Cα range[b]**	**% id[c]**
Geranyltranstransferase*, E. faecalis*	3P8L	8	4,1	130/275	5
Geranylgeranyl pyrophosphate synthase*, S. alba*	2J1P	7,6	3,8	127/277	11
Geranyl pyrophosphate synthase, *M. piperita*	3KRP	7,5	3,9	127/284	10
Polyprenyl pyrophosphate synthase, *A. thaliana*	3AQ0	7,5	3,7	126/312	10
Geranylgeranyl pyrophosphate synthase, *O. sativa*	5XN5	7,5	4,1	129/281	13
Octaprenyl pyrophosphate synthase, *E. coli*	3WJK	7,4	3,7	123/306	11
Trans-isoprenyl pyrophosphate synthase, *C. crescentus*	3OYR	7,4	3,7	126/292	10
Geranylgeranyl pyrophosphate synthase, *A. thaliana*	5E81	7,4	4,2	128/278	13
Geranylfarnesyl pyrophosphate synthase, *A. thaliana*	5E8H	7,4	3,9	127/270	13
Farnesyl pyrophosphate synthase, *B. stearothermophilus*	5AYP	7,3	3,8	124/258	9

[a]Similarity score representing a function that evaluates the overall level of similarity between two structures. *Z*-scores higher than 8.0 indicate that the two structures are most likely homologous (31).

[b]Denotes the number of residues from the query structure that superimpose within an explicit distance cutoff of an equivalent position in the aligned structure.

[c]Denotes the percent sequence identity across the region of structural homology.
