# Supplementary material for: The Loss of Expression of a Single Type 3 Effector (CT622) Strongly Reduces Chlamydia trachomatis Infectivity and Growth
Source: Front Cell Infect Microbiol. 2018 May 15;8:145. doi: 10.3389/fcimb.2018.00145 (PMC5962693; doi:10.3389/fcimb.2018.00145)
Supplement: Table S4 — Primers used. [file Table_4.PDF]

Table S4. List of primers used

Entry vector for CT622 cloning into pDONR207 (Gateway):

CT622f	AAAAAGCAGGCTtgGAAGGAGATAGAACCatggaatcaggaccagaatca
CT622r	AGAAAGCTGGGTcCTAttaagaaagataaccagagaatagagaag

Primers used in a second PCR step to reconstitute the complete *att*B recombinaison sites:

GW1attB1	GGGGACAAGTTTGTACAAAAAAGCAGGCTTG
GW2attB2	GGGGACCACTTTGTACAAGAAAGCTGGGTC

Cloning into pUC19cya for secretion assays in *S. flexneri*:

HindIIICT622	AGTCAAGCTTaaataacggatgtgggctttt
CT622Xba	AGTCTCTAGAattagaagcgatttgcccatt
HindIIICPn0728	AGTCAAGCTTGTAAttgagtttttatggttaatcctattgg
CPn0728Xba	AGTCTCTAGAactcgcctccaatccttgag
HindCCA00015	AGTCAAGCTTGTAAtttttattattatggttaatcctgtcg
CCA00015Xba	AGTCTCTAGAgctcgcctgcatacctaaag

Cloning into pKJ3 for co-expression of His-tagged CT635, Slc1 (CT043) or Mcsc (CT260) and CT622:

BamHICT635	agtcGGATCCaaaaataattccgctcaaaaaattatagattc
CT635stopKpnI	agtcGGTACCttataagggaatccaatttttttcttactt
BamHICT043	agtcGGATCCtccaggcagaatgctgag
CT043StopKpnI	agtcGGTACCttatgcacggattcctgct
BamHICT260	agtcGGATCCacaacgtggactttgaatcaca
CT260StopKpnI	agtcGGTACCctaaggctctagctgatcgga
KpnIRBSCT622	AGTCggtaccAGGAGAtatattgtatggaatcaggaccagaatca
CT622StopSalI	agtcGTCGACttaagaaagataaccagagaatagagaagc
KpnIRBSΔ94CT622	AGTCggtaccAGGAGAtatattgtATgcgagattataatgaggctaaatcgaat

Cloning into pT7HmT of CTL0886:

BamHIΔ94CTL0886	aaaaaaGGATCCGCAAAAGATTATGATAAGGCTAAATCGAATTTTGATACG
CTL0886NotI	ttt ttt GCGGCCGC TTACTGTTTTCTAAACGCATTCTC
CTL0886^{N}NotI	ttt ttt GCGGCCGC TTACAAACCACTAGGTGTGATCGT
BamHICTL0886^{C}	aaa aaa GGATCC TCCTTGTTGCTTGATGATGTAGAC

Cloning of pTTmut9*aadA* and analysis of AS9 clones

CTL0886_447|448s-IBS	AAAAAAGCTTATAATTATCCTTAGAAGACTTAAATGTGCGCCCAGATAGGGTG
CTL0886_447|448sEBS1	CAGATTGTACAAATGTGGTGATAACAGATAAGTCTTAAATAATAACTTACCTTTCTTTGT
CTL0886_447|448s-EBS2	TGAACGCAAGTTTCTAATTTCGATTTCTTCTCGATAGAGGAAAGTGTCT
aadA5	TCTACGCGTTGCCTGACGATGCGTGGAG
GIIR	TCTCGGAGTATACGGCTCTG
Hyp08F	CTCGTAATATGCAAGAGCATTGTAAG
Hyp08R	GGCCGCAGAAGATATTCTGAAG
GIInewF	CGCCCAGATAGGGTGTTAAG
GIInewR	GATTCTCGGCATCGCTTTCGTTTCG
886seqF	GGCAAATCGCTTCTAATTCGGAAAC
886seqR	CGCAGGAATCTGATCCGCAGAGTCG
pJETF	CGACTCACTATAGGGAGAGCGGC
pJETR	AAGAACATCGATTTTCCATGGCAG

Primers used for qPCR

euo	TATGCTACACGCATTGGTGCT and GCCTCAAAACCTTCTCTCATGG
omcB	CTGCAACAGTATGCGCTTGTC and GCTGTTGCTGTTCCTTGGTTC
hctA	ATGGCGCTAAAAGATACGGC and TGTGCTGCGGCTTTATTTCC
