# Supplementary material for: The Loss of Expression of a Single Type 3 Effector (CT622) Strongly Reduces Chlamydia trachomatis Infectivity and Growth
Source: Front Cell Infect Microbiol. 2018 May 15;8:145. doi: 10.3389/fcimb.2018.00145 (PMC5962693; doi:10.3389/fcimb.2018.00145)
Supplement: Table S5 — Statistical analyses. [file Table_5.PDF]

Figure	Comparison	Alternative hypothesis	Sample size	Test	*P*-value
4A	WT and AS9	Median difference	$n_{WT} = 4$, $n_{AS9} = 4$	Mann-Whitney-Wilcoxon test	0.03
4B	WT and AS9complCT622	Median difference	$n_{WT} = 3$, $n_{AS9complCT622} = 3$	Mann-Whitney-Wilcoxon test	0.10
4C	WT and AS9 in 30 min	Difference of effect	$n_{WT} = 5$, $n_{AS9} = 3$, df = 30	Contrast test in linear model	0.001
	WT and AS9complCT622 in 30 min	Difference of effect	$n_{WT} = 5$, $n_{AS9complCT622} = 3$, df = 30	Contrast test in linear model	0.006
	AS9 and AS9complCT622 in 30 min	Difference of effect	$n_{AS9} = 3$, $n_{AS9complCT622} = 3$, df = 30	Contrast test in linear model	0.53
	WT and AS9 in 1h	Difference of effect	$n_{WT} = 4$, $n_{AS9} = 4$, df = 30	Contrast test in linear model	0.001
	WT and AS9complCT622 in 1h	Difference of effect	$n_{WT} = 4$, $n_{AS9complCT622} = 3$, df = 30	Contrast test in linear model	0.04
	AS9 and AS9complCT622 in 1h	Difference of effect	$n_{AS9} = 4$, $n_{AS9complCT622} = 3$, df = 30	Contrast test in linear model	0.27
	WT and AS9 in 2h	Difference of effect	$n_{WT} = 4$, $n_{AS9} = 4$, df = 30	Contrast test in linear model	0.001
	WT and AS9complCT622 in 2h	Difference of effect	$n_{WT} = 4$, $n_{AS9complCT622} = 3$, df = 30	Contrast test in linear model	0.10
	AS9 and AS9complCT622 in 2h	Difference of effect	$n_{AS9} = 4$, $n_{AS9complCT622} = 3$, df = 30	Contrast test in linear model	0.10
5A	WT and AS9	Median difference	$n_{WT} = 26$, $n_{AS9} = 26$	Mann-Whitney-Wilcoxon test	1e-6
	WT and AS9complCT622	Median difference	$n_{WT} = 26$, $n_{AS9complCT622} = 26$	Mann-Whitney-Wilcoxon test	0.01
	AS9 and AS9complCT622	Median difference	$n_{AS9} = 26$, $n_{AS9complCT622} = 26$	Mann-Whitney-Wilcoxon test	3e-5
6B	WT and AS9 in 1h in euo	Difference of effect	$n_{WT} = 6$, $n_{AS9} = 6$, df = 30	Contrast test in linear model	5e-5
	WT and AS9 in 3h in euo	Difference of effect	$n_{WT} = 6$, $n_{AS9} = 6$, df = 30	Contrast test in linear model	7e-4
	0h and 1h in WT in euo	Difference of effect	$n_{0h} = 6$, $n_{1h} = 6$, df = 30	Contrast test in linear model	1e-4
	1h and 3h in WT in euo	Difference of effect	$n_{1h} = 6$, $n_{3h} = 6$, df = 30	Contrast test in linear model	0.46
	0h and 1h in AS9 in euo	Difference of effect	$n_{0h} = 6$, $n_{1h} = 6$, df = 30	Contrast test in linear model	0.75
	1h and 3h in AS9 in euo	Difference of effect	$n_{1h} = 6$, $n_{3h} = 6$, df = 30	Contrast test in linear model	0.85
	WT and AS9 in 1h in omcB	Difference of effect	$n_{WT} = 6$, $n_{AS9} = 6$, df = 30	Contrast test in linear model	0.94
	WT and AS9 in 3h in omcB	Difference of effect	$n_{WT} = 6$, $n_{AS9} = 6$, df = 30	Contrast test in linear model	0.19
	0h and 1h in WT in omcB	Difference of effect	$n_{0h} = 6$, $n_{1h} = 6$, df = 30	Contrast test in linear model	3e-7
	1h and 3h in WT in omcB	Difference of effect	$n_{1h} = 6$, $n_{3h} = 6$, df = 30	Contrast test in linear model	0.50
	0h and 1h in AS9 in omcB	Difference of effect	$n_{0h} = 6$, $n_{1h} = 6$, df = 30	Contrast test in linear model	3e-7
	1h and 3h in AS9 in omcB	Difference of effect	$n_{1h} = 6$, $n_{3h} = 6$, df = 30	Contrast test in linear model	0.05
	WT and AS9 in 1h in hctA	Difference of effect	$n_{WT} = 6$, $n_{AS9} = 6$, df = 30	Contrast test in linear model	0.11
	WT and AS9 in 3h in hctA	Difference of effect	$n_{WT} = 6$, $n_{AS9} = 6$, df = 30	Contrast test in linear model	0.59
	0h and 1h in WT in hctA	Difference of effect	$n_{0h} = 6$, $n_{1h} = 6$, df = 30	Contrast test in linear model	3e-5
	1h and 3h in WT in hctA	Difference of effect	$n_{1h} = 6$, $n_{3h} = 6$, df = 30	Contrast test in linear model	0.63
	0h and 1h in AS9 in hctA	Difference of effect	$n_{0h} = 6$, $n_{1h} = 6$, df = 30	Contrast test in linear model	3e-7
	1h and 3h in AS9 in hctA	Difference of effect	$n_{1h} = 6$, $n_{3h} = 6$, df = 30	Contrast test in linear model	0.12

[a] n, sample size for each class tested; df, degree of freedom; NA, not adapted (no multiple comparisons); adjusted p values from the same panel, according to Benjamini & Hochberg
