# Supplementary figures and images for: The Loss of Expression of a Single Type 3 Effector (CT622) Strongly Reduces Chlamydia trachomatis Infectivity and Growth
Source: Front Cell Infect Microbiol. 2018 May 15;8:145. doi: 10.3389/fcimb.2018.00145 (PMC5962693; doi:10.3389/fcimb.2018.00145)

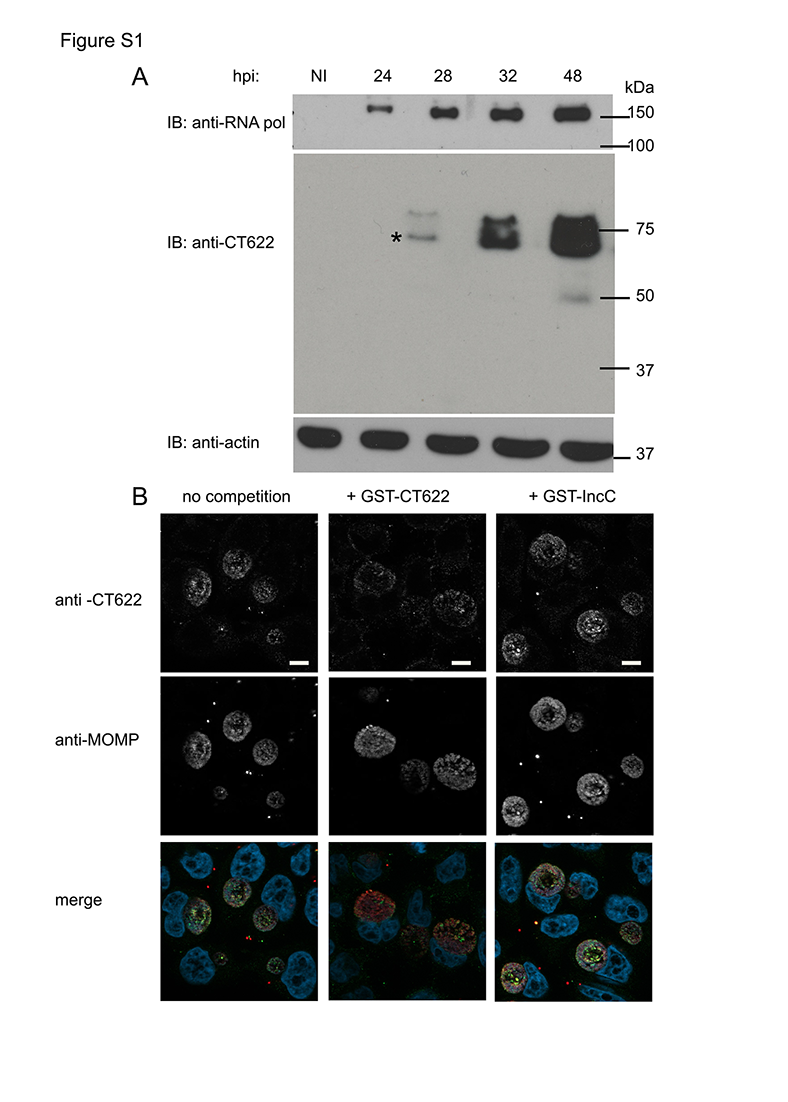

Supplement: Figure S1 — Detection of CT622 with rabbit polyclonal antibody. (A) HeLa cells were infected with LGV L2 at MOI = 0.5. At the indicated time, cells were collected and total cell lysate were prepared in urea buffer. Proteins were resolved in 8% polyacrylamide gels and probed by immunoblotting with antibodies to CT622, bacterial RNA polymerase, or actin. The expected molecular weight of CT622 is 68 kDa (asterisk). A cross-reaction product around 78 kDa was also detected by anti-CT622 antibodies. NI, not infected. (B) HeLa cells were infected with LGV L2 for 24 h before fixation and permeabilization. Purified anti-CT622 antibodies (0.5 μg/ml) were preincubated or not for 30 min with an excess (10 μg/ml) of the indicated recombinant protein before contact with the coverslip. Bacteria were labeled with mouse anti-MOMP antibodies. Secondary antibodies used were Alexa488-conjugated anti-rabbit antibodies (green) and Cy5-conjugated anti-mouse antibodies (red). DNA was stained with Hoechst (blue in merge picture). Most of the anti-CT622 signal disappeared in the presence of an excess of GST-CT622 and not of an irrelevant protein. A faint signal remained, likely corresponding to the bacterial cross-reaction product observed by western blot. Scale bar = 10 μm. [file Image_1.TIF]

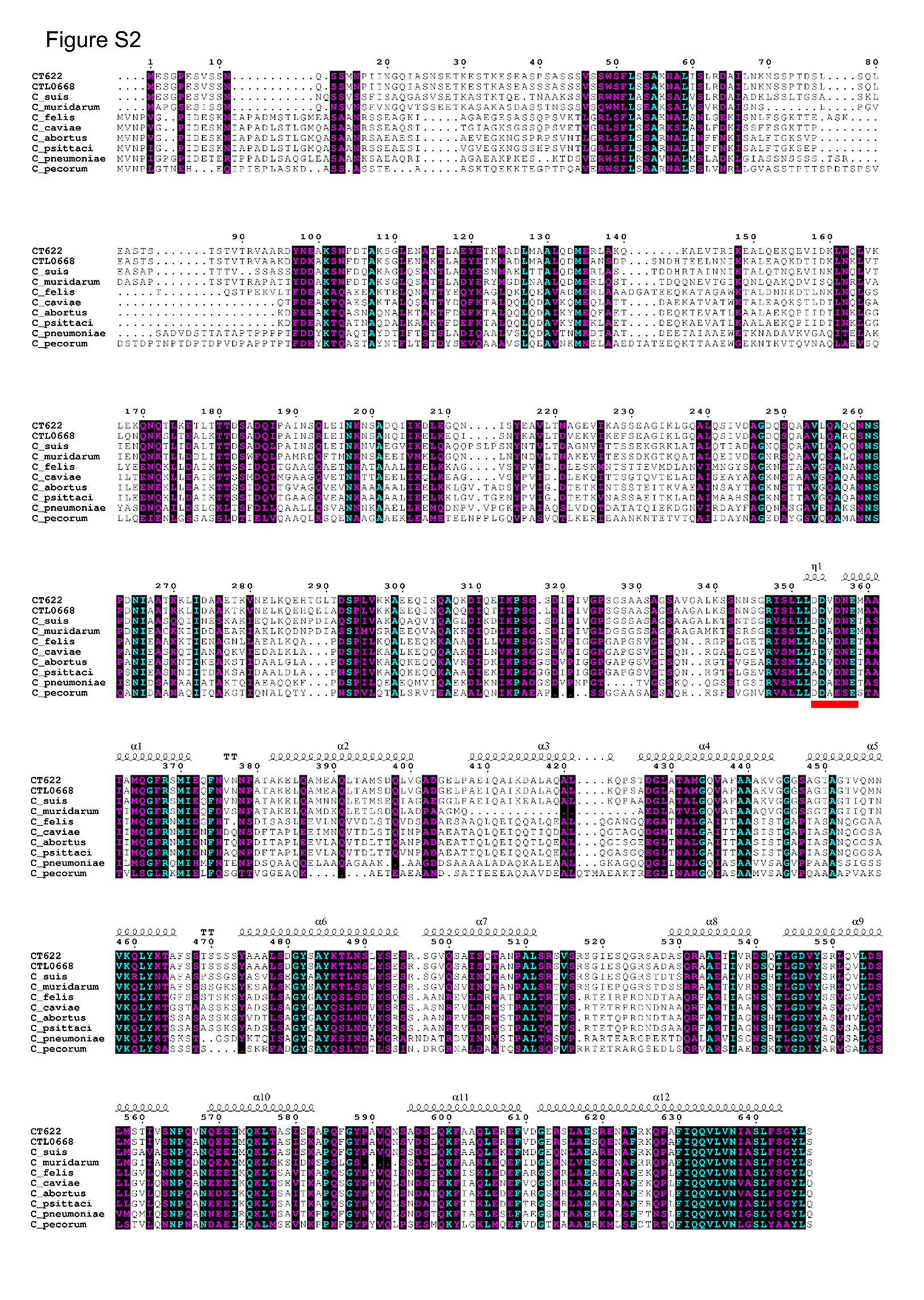

Supplement: Figure S2 — Multiple Sequence Alignment of CT622 and Orthologs from Chlamydiaceae. Sequence alignment was generated using ClustalW and rendered with ESPRIPT. The numbers above the sequences correspond to C. trachomatis CTL0886. The secondary structure of CTL0886C is shown above the alignment. Residues are colored according to conservation (cyan, identical, and purple, similar) as judged by the BLOSUM62 matrix. Red bar highlights stretch of negatively charged side chains. [file Image_2.TIF]

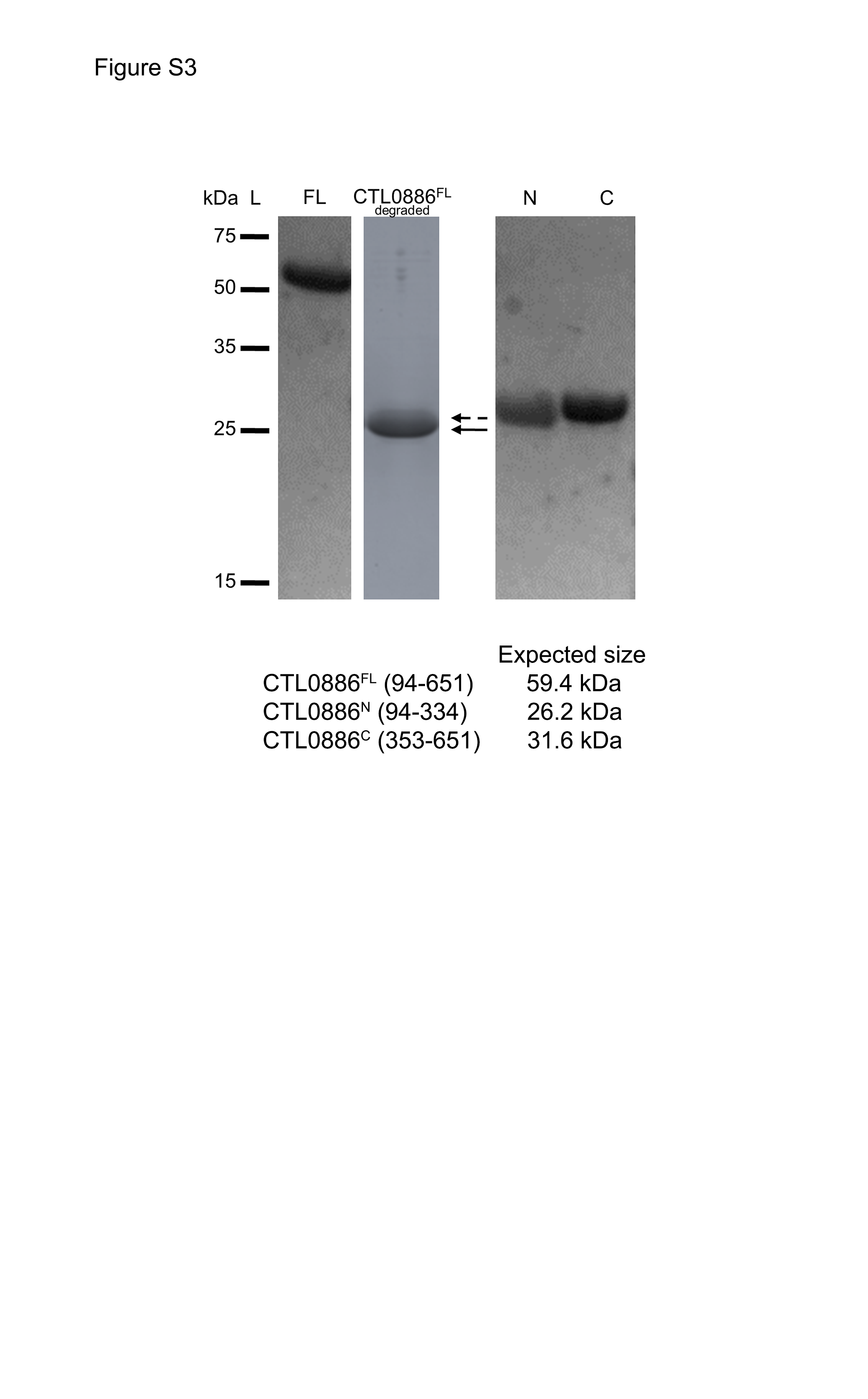

Supplement: Figure S3 — Purification of Recombinant CTL0886. Purified CTL0886FL (residues 94–651) as well as a degraded full-length sample were analyzed by SDS-PAGE. Two arrows indicate the presence of overlapping protein bands in the degraded sample. LC-MS/MS analysis indicated they contained residues 94–334 (dashed arrow) and 353–651 (solid arrow). These fragments were subsequently expressed and purified, CTL0886N (residues 94–322) and CT0886C (residues 353–651). [file Image_3.TIF]

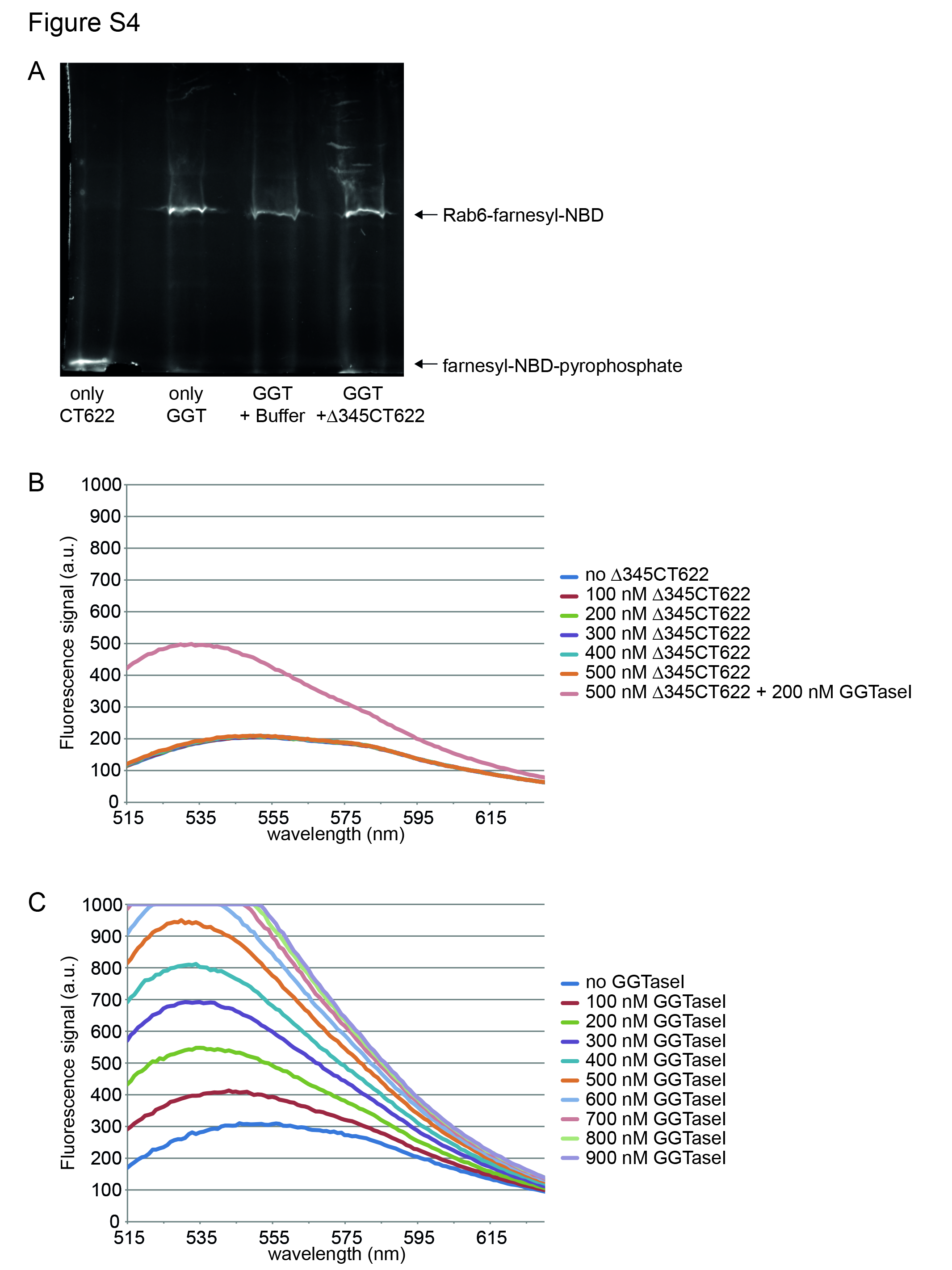

Supplement: Figure S4 — Functional assays on CT622 activity regarding prenylation reactions. (A) Fluorescence scan to reveal farnesyl-NBD fluorescence on an SDS-PAGE of protein samples after prenylation tests. In the left two lanes it was tested whether GST-CT622 can prenylate Rab6 in the presence of Rab Escort Protein. GST-CT622 (only CT622) does not prenylate Rab6 whereas the positive control with RabGGTase (only GGT) shows a clear band with prenylated protein. 0.5 nmol of RabGGTase (GGT) or GST-CT622 (CT622) were used. In the right two lanes 2.5 nmol GST-Δ345CT622 was added to a prenylation reaction with 0.5 nmol RabGGTase to test whether CT622 can inhibit prenylation by RabGGTase by competitive binding (GGT+-Δ345CT622). No inhibition was observed. (B) Fluorescence spectrometry measurements of a titration to test binding of farnesyl-NBD-pyrophosphate (500 nM) to GST-Δ345CT622. No fluorescence change can be observed upon addition of GST-Δ345CT622. As a final step 200 nM GGTaseI was added to show fluorescence increase upon nucleotide binding. (C) Positive control: exemplary fluorescence spectrometry measurements of a titration with GGTaseI α+β to show farnesyl-NBD fluorescence change (650 nM) upon protein binding. [file Image_4.TIF]
